# Reconciling seascape genetics and fisheries science in three codistributed flatfishes

**DOI:** 10.1111/eva.13139

**Published:** 2020-11-02

**Authors:** Sara Vandamme, Joost A. M. Raeymaekers, Gregory E. Maes, Karl Cottenie, Federico C. F. Calboli, Eveline Diopere, Filip A. M. Volckaert

**Affiliations:** ^1^ Laboratory of Biodiversity and Evolutionary Genomics KU Leuven Leuven Belgium; ^2^ Animal Sciences Unit ‐ Fisheries and Aquatic Production Flanders Research Institute for Agriculture, Fisheries and Food (ILVO) Oostende Belgium; ^3^ Department of Animal Sciences and Aquatic Ecology Ghent University Oostende Belgium; ^4^ Faculty of Biosciences and Aquaculture Nord University Bodø Norway; ^5^ Centre for Sustainable Tropical Fisheries and Aquaculture Comparative Genomics Centre College of Sciences and Engineering James Cook University Townsville QLD Australia; ^6^ Center for Human Genetics Genomics Core KU Leuven Leuven Belgium; ^7^ Department of Integrative Biology University of Guelph Guelph ON Canada; ^8^ CeMEB Department of Marine Sciences University of Gothenburg Gothenburg Sweden

**Keywords:** conservation genetics, continental shelf, fisheries management, flatfish, population genetics, redundancy analysis, seascape genetics

## Abstract

Uncertainty hampers innovative mixed‐fisheries management by the scales at which connectivity dynamics are relevant to management objectives. The spatial scale of sustainable stock management is species‐specific and depends on ecology, life history and population connectivity. One valuable approach to understand these spatial scales is to determine to what extent population genetic structure correlates with the oceanographic environment. Here, we compare the level of genetic connectivity in three codistributed and commercially exploited demersal flatfish species living in the North East Atlantic Ocean. Population genetic structure was analysed based on 14, 14 and 10 neutral DNA microsatellite markers for turbot, brill and sole, respectively. We then used redundancy analysis (RDA) to attribute the genetic variation to spatial (geographical location), temporal (sampling year) and oceanographic (water column characteristics) components. The genetic structure of turbot was composed of three clusters and correlated with variation in the depth of the pycnocline, in addition to spatial factors. The genetic structure of brill was homogenous, but correlated with average annual stratification and spatial factors. In sole, the genetic structure was composed of three clusters, but was only linked to a temporal factor. We explored whether the management of data poor commercial fisheries, such as in brill and turbot, might benefit from population‐specific information. We conclude that the management of fish stocks has to consider species‐specific genetic structures and may benefit from the documentation of the genetic seascape and life‐history traits.

## 1 INTRODUCTION

Worldwide fish stocks managed properly with the best scientific evidence available are either rebuilding or consistently above target levels (FAO, 2020). Stocks solely defined as geopolitical, geographical or management units without consideration of biologically relevant information are prone to failure (Hauser & Carvalho, 2008; Pita et al., 2016; Reiss et al., 2009). Two problems are associated with the mismatch between geographical and biological units. First, genetically homogenous populations may cover several management zones, each assessed independently, which may result in unnecessarily small quota and localized management (Ovenden et al., 2015; Roy et al., 2012). Second, genetically discrete populations may have overlapping distributions at various life stages within a single management zone. In such circumstances, less productive populations are more susceptible to local overharvesting or even extinction (Reiss et al., 2009; Roy et al., 2012). Furthermore, even within a seemingly homogenous stock, individual variability in life‐history and behavioural strategies may further constrain the usefulness of the stock unit (Hauser & Carvalho, 2008; Pita et al., 2016; Reiss et al., 2009). Relatively small losses of genetic variability may have irreversible consequences on the functional role of species within the ecosystem and their long‐term viability. These small losses may represent unique genetic combinations that support the capacity of populations to adapt to contrasting environmental conditions or environmental change (Dann et al., 2013; Roy et al., 2012; Scheffers et al., 2016; Schindler et al., 2010). Knowledge of population genetic structure and its determinants is therefore fundamental to identify resilient populations under continuous harvesting (Dann et al., 2013; Reiss et al., 2009).

Populations are an integral part of communities and ecosystems. Ecosystem‐based management (EBM) includes key principles such as ecosystem connections, appropriate spatial and temporal scales, use of scientific knowledge, ecological integrity and biodiversity, and sustainability (Long et al., 2015). Hence, EBM incorporates the full dimensions of biodiversity, namely populations, species and ecosystems as specified in the United Nations Convention on Biological Diversity (CBD; www.cbd.int) signed in 1992. The holistic approach of EBM has gained considerable momentum in the fisheries sector. Ecosystem‐based fisheries management (EBFM) aims at sustaining healthy marine ecosystems and the fisheries they support (Pikitch et al., 2004). The merits of a multispecies approach, which is an integral part of EBFM, is that co‐occurring species, captured simultaneously, are managed simultaneously despite their distinct biological properties. Cases of comanaged fish communities and ecosystems are found worldwide (Morales‐Nin et al., 2017; Rochet et al., 2011; Thorpe, 2019). Our paper incorporates multispecies genetic biodiversity by comparing the genetic seascape of three codistributed demersal flatfishes across the North East Atlantic Ocean.

Coupling stock‐specific environmental and biological knowledge with genetic population structure within and across existing management areas is important to delineate scales and specify subareas for fisheries management (Fogarty & Botsford, 2007; Pita et al., 2016). However, uncovering spatial genetic heterogeneity in the ocean is challenging because of the high level of connectivity and the large size of the populations (Hauser & Carvalho, 2008). It is accepted that population divergence may occur in the face of gene flow in an open marine environment (Pinho & Hey, 2010). Connectivity (realized gene flow) is dynamically shaped by spatial heterogeneity of the oceanographic conditions, larval behaviour and adult reproductive biology (Mora & Sale, 2002; Selkoe et al., 2016). The direction and variability of oceanographic currents allow for larval advection but also retention (Sinclair & Power, 2015), with a spatial scale largely determined by the physical characteristics of the system (Riginos et al., 2016). At the same time, larvae modulate selective transport through behavioural adaptation (Morgan & Fisher, 2010). Adult reproduction, such as the timing, duration and site selection of spawning, is also tightly linked with the environment (Riginos & Liggins, 2013; Selkoe et al., 2016). Additionally, the large effective population sizes lower population discreteness (Hauser & Carvalho, 2008; Waples, 1998).

Comparative frameworks provide excellent insights on the processes of population divergence and speciation (Gagnaire, 2020; Raeymaekers et al., 2017). Relatively few comparative marine studies have been published, either as a meta‐analysis (Jenkins et al., 2018; Pascual et al., 2017; Patarnello et al., 2007) or as original research (Le Moan et al., 2019; Stanley et al., 2018). In this study, we investigate the merits of a comparative seascape genomic approach among three commercially exploited fishes. Turbot (*Scophthalmus maximus*), brill (*S. rhombus*) and sole (*Solea solea*) live on the continental shelves of the North East Atlantic Ocean from Norway to the Iberian Peninsula—brill and sole to North Africa, the Mediterranean Sea and Black Sea. Turbot also breeds in the brackish Baltic Sea. These species represent two families (Scophthalmidae and Soleidae) of temperate marine flatfishes, each with distinct life‐history traits (LHT), but sharing the same seascape. Turbot matures at a determinate moment and spawns during a short spawning moment (so‐called capital spawner). Brill matures over a protracted period and has an intermittent release of eggs, which is characteristic for an income spawner. Both spawn offshore; eggs and larvae have pelagic phases of short to intermediate duration (van der Hammen et al., 2013). Sole is also income spawner, with a spawning time that varies with latitude and overlaps with brill in the North Sea. Its spawning grounds are located offshore in the southern range (Amara et al., 2000) and inshore in the northern range (Rijnsdorp et al., 1992). Spawning time in the northern range varies from early spring (brill: March–July, peak spawning in May; sole: February–May, peak spawning in April) to late summer (turbot: April–August, peak spawning in May–June) (Table 1). All three species share the same shallow nursery grounds along the coasts and estuaries of the North East Atlantic Ocean. Adult sole feed on macrobenthos at night, while turbot and brill feed on larger sized prey, such as fish and crustaceans, during day time.

**TABLE 1:** Overview of the main life history traits of the demersal flatfish turbot *Scophthalmus maximus,* brill *Scophthalmus rhombus* and sole *Solea solea* in the North Sea

Life‐history Trait	Species		
	*Scophthalmus maximus*	*Scophthalmus rhombus*	*Solea solea*
	Turbot	Brill	Sole
Adult depth distribution	70–80 m^1,2^	70–80 m^1,2^	Mainly < 50 m^3,4^
Spawning location	Offshore^5,6,7^	Offshore^5,6,7^	Inshore and restricted^3,8^
Spawning time	April–August^7,9^	March–July^7,9^	February–May^3,8^
Peak spawning	May–June	May	April
Adult density at spawning site^7^	1 per 2 × 10^6^ m^2^	1 per 2 × 10^6^ m^2^	4 per 10^4^ m^2^
Nursery location	Shallow coastal waters (1 m)^10,11,12^	Shallow coastal waters (1 m)^10,11,12^	Shallow coastal waters^3,12^
Larval duration	Pelagic larval phase of 68 days^13,14^	Pelagic larval phase of 2 months ^13^	Pelagic larval phase of 1 month^3^
Type of spawner	Capital spawner: spawns during a short spawning moment	Income spawner: intermittent release of eggs	Income spawner: intermittent release of eggs
Average fecundity (eggs/g)^3^	1,078	465	800
Egg size (mm)^3^	0.9–1.2	1.28–1.65	1.0–1.6
Settling (mm)^3^	23–39	25	7–10

Note

1. Déniel (1981), 2. Felix, Vinagre & Cabral (2011) and references therein, 3. Rijnsdorp et al. (1992), 4. Gibson, Nash, Geffen & van der Veer (2015), 5. Rae & Devlin (1972), 6. Delbare & De Clerck (1999), 7. van der Hammen et al. (2013), 8. Lacroix, Maes, Bolle & Volckaert (2013), 9. Jones (1974), 10. Riley, Symonds & Woolner (1981), 11. Gibson (1997), 12. Beyst, Buysse, Dewicke & Mees (2001), 13. Jones (1972), 14. Nissling, Johansson & Jacobsson (2006)

Regardless of their overlapping distribution ranges, these three species exhibit different genetic population substructuring patterns. Two distinct populations and a pattern of isolation by distance in the Atlantic population characterize the neutral genetic structure of sole. A comparison of northern and southern Atlantic populations suggests adaptation to temperature (Cuveliers et al., 2012; Diopere et al., 2018). Outlier loci separate a fourth population in the Irish and Celtic Sea (Diopere et al., 2018). Three populations of turbot live in the North East Atlantic Ocean (Atlantic, Western Irish Shelf and Baltic Sea). Furthermore, turbot in the North Sea differ subtly between the northern and southern region (Prado, et al., 2018; Vandamme et al., 2014). Based on allozymes, brill reveals almost no genetic structure (Blanquer et al., 1992). Our study of brill is the first in 25 years to reassess the genetic structure throughout its northern range. While sole is a well‐documented target species of the European demersal fishery, brill and turbot are poorly documented by‐catch of a mixed fishery (van der Hammen et al., 2013; Kerby et al., 2013). Hence, fishery‐dependent and ‐independent catch records are scarce for these two species, which reduces the reliability of stock assessment. Moreover, stock management areas of all three species differ without any clear biological justification throughout their northern range. Failure to assign appropriate management measures raises doubts on the appropriateness of currently designated management units (Ovenden et al., 2015; Reiss et al., 2009; Schindler et al., 2010).

Our comparative seascape genetic study aims at identifying the environmental and spatio‐temporal determinants of population genetic connectivity in these three codistributed flatfishes. To do so, we target the same biogeographical region, environmental data and biostatistical tools to characterize the shelf populations of each species. In addition, we apply a similar sampling design (similar distribution of sampling sites and sample size) and the same type of molecular markers (microsatellites). We hypothesize that populations of codistributed and ecologically similar flatfishes are structured at comparable environmental and spatio‐temporal scales. We discuss the observed subtle genetic structure of each species and the interaction between hydrodynamics and larval dispersal, and the role of adult dispersal. We explore the results in the context of an integrated framework for fish stock management.

## 2 MATERIALS AND METHODS

### 2.1 Study design and sampling

**FIGURE 1:**
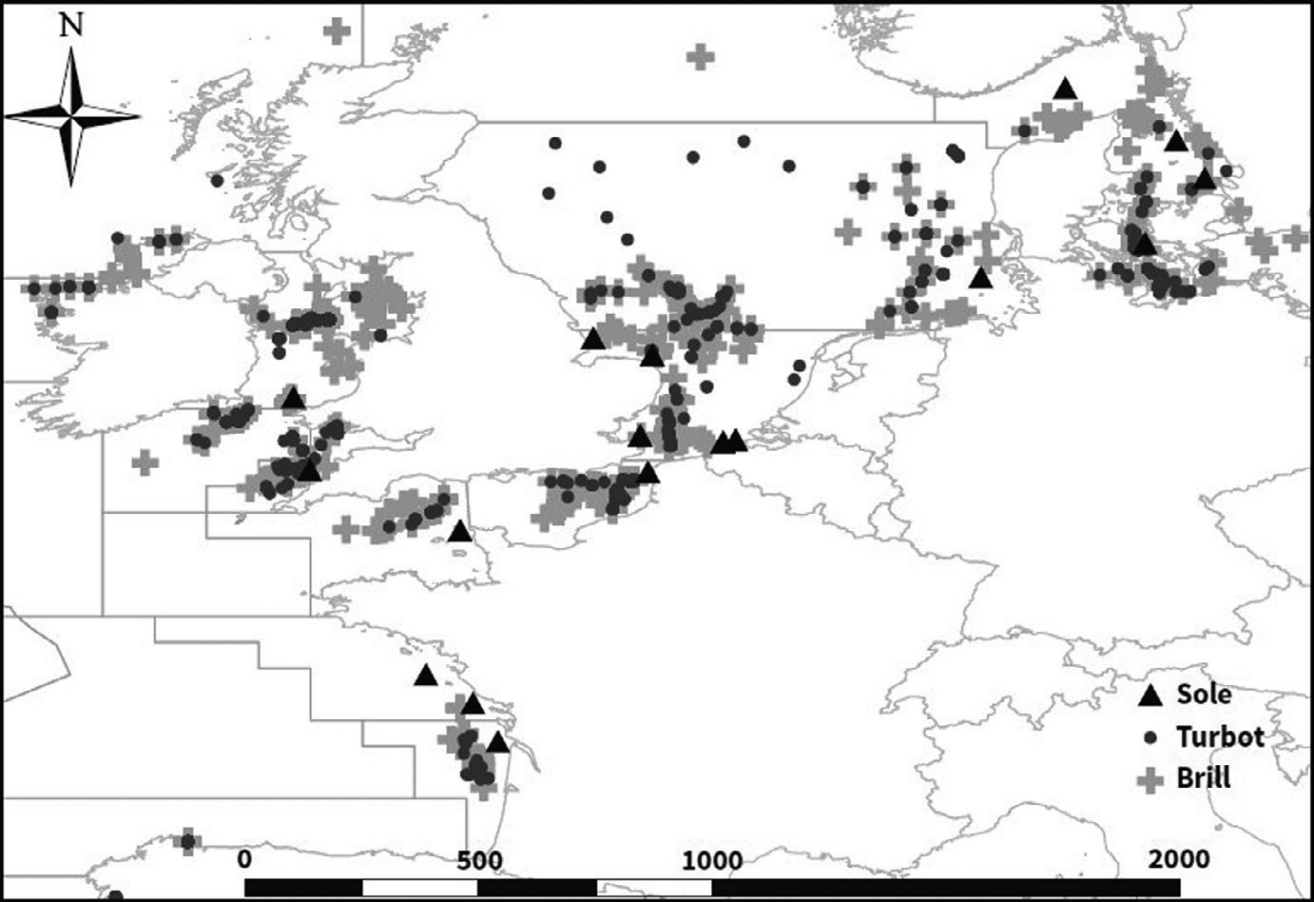
Sampling sites of turbot, brill and sole in the North East Atlantic Ocean

This study combines newly generated genotypes for brill with existing genotypes for turbot (Vandamme et al., 2014) and sole (Cuveliers et al., 2012). Yet, all data are derived from samples collected by beam trawling during field surveys on board of research vessels or commercial vessels across the North East Atlantic Ocean. Specifically, we targeted the geographical regions where the three species co‐occur. During the surveys, fin‐clip samples were collected from adult individuals. Surveys were conducted in the year 2000 and from 2006 to 2010 (Table 2). We therefore could investigate both the spatial and a short‐term temporal scale of population genetic differentiation. Up to three years of samples from the same locations were available for turbot and brill. In order to restrict the comparison between turbot and brill geographically, a selection of the turbot samples available from Vandamme et al. (2014) was included. Sole samples were grouped in “populations” based on their catch location coinciding with the same ICES (International Council for the Exploration of the Sea) areas as brill and turbot. In total, 23 samples from 14 sites for turbot (*N* = 748), 23 samples from 15 sites for brill (*N* = 879) and 13 samples from 11 sites for sole (*N* = 1,125) were considered (Figure 1; Table 2).

**TABLE 2:** Sampling information for turbot, brill and sole including latitude (Lat), longitude (Long), sample (area × year), and sample size (*N*). Estimates of genetic diversity include expected and observed heterozygosity (*H*_e_ and *H*_o_, respectively), allelic richness (AR) and inbreeding coefficient (*F*_IS_). Significant *F*_IS_ values are in bold

Geographical region	Sample location	Turbot							Brill												Sole															
		Mean position		Sample		N	He	Ho	AR	F_IS_	Sample		N		He		Ho		AR		F_IS_		Sample			N			He			Ho			AR			F_IS_		
				Lat	Long						Area	Year	Area	Year												Area	Year														
Transition area	Belt Sea	54.5	11.2	BEL	2010	39	0.654	0.643	5.37	0.032	BEL	2010		16		0.759		0.769		6.87		0.032																		
		55.9	11.3	BEL	2009	26	0.667	0.623	6.16	**0.068**	BEL	2009		38		0.781		0.715		7.25		**0.100**		BEL	2007			40			0.735			0.740			8.66	−0.010		
	Kattegat	57.0	11.3	KAT	2009	15	0.642	0.642	5.81	0.053	KAT	2009		30		0.759		0.728		7.05		**0.058**		KAT	2007			71			0.746			0.732			8.33	0.006		
	Skagerrak	58.2	11.0								SKR	2009		17		0.768		0.754		7.24		0.048		SKR	2007			24			0.749			0.717			8.71	0.026		
North Sea	German Bight	55.5	6.7	ENS	2010	53	0.655	0.630	5.75	0.046	ENS	2010		25		0.743		0.670		6.88		**0.125**																		
		53.8	6.5								ENS	2009		15		0.752		0.751		7.57		0.033		ENS	2007			33			0.757			0.685			8.68	**0.093**		
	Central North Sea	54.7	2.1	CNS	2010	14	0.655	0.684	5.43	−0.009														CNS	2008			39			0.752			0.733			9.07	0.022		
		51.7	2.2	CNS	2007	48	0.649	0.662	5.53	−0.012	CNS	2007		66		0.764		0.739		7.01		**0.045**		CNS	2007			20			0.757			0.715			8.50	0.052		
	Southern North Sea	52.5	1.9	SNS	2007	18	0.621	0.563	5.51	**0.126**														SNS	2007			277			0.771			0.725			9.07	**0.055**		
		51.8	1.8	SNS	2009	32	0.666	0.642	5.70	**0.064**	SNS	2009		42		0.761		0.751		6.85		0.017		SNS	2008			171			0.760			0.702			9.09	**0.077**		
		52.2	2.4								SNS	2010		33		0.761		0.747		6.99		0.040																		
English Channel	Eastern English Channel	50.4	0.2	EEC	2007	29	0.647	0.648	5.47	0.017	EEC	2007		37		0.777		0.745		7.09		**0.057**																		
		50.5	1.1	EEC	2009	51	0.674	0.658	5.91	0.029	EEC	2009		66		0.774		0.743		7.12		**0.042**		EEC	2008			45			0.753			0.700			8.87	**0.067**		
		50.0	−0.2								EEC	2010		44		0.778		0.744		7.10		**0.060**																		
	Western English Channel	50.0	−2.8	WEC	2010	16	0.649	0.634	5.81	0.052	WEC	2010		35		0.777		0.745		6.99		**0.051**		WEC	2009			74			0.749			0.703			8.86	**0.055**		
British Isles	Bristol Channel	50.7	−5.5	BCH	2007	16	0.644	0.674	5.53	−0.016	BCH	2007		29		0.772		0.691		7.03		**0.118**																		
		51.4	−4.7	BCH	2009	20	0.675	0.661	5.90	0.049	BCH	2009		17		0.763		0.743		7.19		0.064		BCH	2008			72			0.748			0.739			8.74	−0.003		
		50.8	−5.5	BCH	2010	43	0.662	0.634	5.64	**0.049**																														
	South East Ireland	51.6	−6.0	SEI	2009	90	0.673	0.645	5.85	0.044	SEI	2009		78		0.770		0.721		6.91		**0.071**																		
	Irish Sea	53.5	−53.0	IRS	2006	21	0.630	0.575	5.44	**0.099**																														
		53.6	−5.0	IRS	2007	20	0.625	0.642	5.63	−0.007	IRS	2007		59		0.766		0.738		7.09		0.043																		
		53.6	−5.0	IRS	2009	82	0.678	0.677	5.76	0.002	IRS	2009		87		0.780		0.731		7.32		**0.076**		IRS	2008			88			0.766			0.759			9.18	0.001		
	West Ireland	54.6	−9.0	WIR	2009	26	0.672	0.670	5.76	0.022	WIR	2009		29		0.780		0.797		7.30		0.011																		
	West Scotland	55.4	−7.7								WSC	2009		19		0.759		0.764		7.18		−0.005																		
Iberian Peninsula	Bay of Biscay	45.6	−2.1								BOB	2006		18		0.770		0.756		6.79		0.057																		
		45.2	−1.8	BOB	2007	25	0.652	0.600	5.84	**0.102**	BOB	2007		49		0.777		0.759		7.34		**0.029**		BOB	2007			171			0.761			0.725			8.97	**0.042**		
		45.2	−1.8	BOB	2009	18	0.680	0.673	5.47	0.044																													
	North and North West Spain	43.7	−7.4	NWS	2000	27	0.668	0.661	5.64	0.040	NWS	2000		30		0.763		0.747		6.98		**0.033**																	
	Portuguese Coast	42.6	−8.8	POR	2000	19	0.668	0.677	5.83	0.019																														

### 2.2 DNA extraction and microsatellite genotyping

For brill, DNA was extracted with the NucleoSpin Tissue extraction kit (Macherey Nagel GmBH). Brill samples were genotyped at 14 microsatellite loci, including two markers described in Iyengar et al. (2000) (*Sma5‐111INRA* and *SmA1‐152INRA*), two EST‐derived markers described in Bouza et al. (2008) (*SmaUSC‐E32* and *SmaUSC‐E41*) and 10 novel markers developed through gDNA pyrosequencing (GS FLX Titanium, Roche; *ScoR26*, *ScoR28*, *ScoR12*, *ScoR27*, *ScoR16*, *ScoR5*, *ScoR2*, *ScoR11*, *ScoR4* and *ScoR6*) (Molecular Ecology Resources Primer Development et al., 2012). Microsatellite markers were combined in three multiplex reactions, each consisting of an initial denaturation step of 7 min at 95°C, followed by 30 cycles of 30 s at 95°C, 90 s at 54°C (multiplex 2 and 3) or 56°C (multiplex 1) and 60 s at 72°C after a final elongation of 30 s at 60°C, and cooled down to 10°C. Fragment analysis was performed on an ABI 3130 AVANT Genetic Analyzer (Applied Biosystems) using GeneScan‐500 LIZ internal lane size standard. Allele sizes were determined using Genemapper v4.0 (Applied Biosystems). The TANDEM v1.07 software was used for automated allele binning (Matschiner et al., 2009). For turbot and sole, similar procedures for DNA extraction and genotyping were used as described here for brill. In short, genotypes for 14 noncoding microsatellites were generated for turbot by Vandamme et al. (2014) and for 10 noncoding microsatellite markers for sole by Cuveliers et al. (2012).

### 2.3 Population genetics

Genotypes were analysed in order to compare levels of genetic variation and genetic differentiation between the three species. Multilocus genotypes were tested for deviations from Hardy–Weinberg equilibrium using the pegas package in the R software (Paradis, 2010; R Core Team, 2020). Linkage disequilibrium was evaluated using Fisher's exact test implemented in the genepop package in R (Rousset, 2008). R package hierfstat was used to test for significance of *F*_IS_ (reflecting heterozygote deficiency/excess) using a randomization test (Goudet & Jombart, 2015). Subsequently, the level of genetic variation for each sample was estimated as number of alleles (allelic richness), observed (*H*_O_) and expected (*H*_E_) heterozygosity. Genetic structure among populations within each species was investigated using two methods. First, population structure in space and time was investigated using global and pairwise *F*_ST_ between all samples (using Weir & Cockerham, 1984 statistics) using FSTAT. A correction for multiple testing used the method of Benjamini and Hochberg (1995) through control of the false discovery rate (FDR). Nonmetric multidimensional scaling (NMDS) analysis of the *F*_ST_ values among samples was done using isoMDS from the mass package in R (Venables & Ripley, 2002). Second, individual genotypes were clustered with a Bayesian algorithm using a nonadmixture model, correlated frequency and spatio‐temporal origin as prior information (STRUCTURE v2.3.4; (Pritchard et al., 2000)). Such ancestry model was suggested because of the lack of prior knowledge on the origin of the populations under study (Porras‐Hurtado et al., 2013). For each simulation of *K* (1–6), 10 independent replicates were run. For each replicate, 10^4^ iterations were used as burn‐in, followed by 10^5^ Markov Chain Monte Carlo (MCMC) iterations. At completion of the STRUCTURE runs, the most likely number of clusters was selected by choosing *K* with the largest log‐likelihood (Evanno et al., 2005), implemented in the STRUCTURE HARVESTER v0.6.94 web application (Earl & vonHoldt, 2012).

### 2.4 Seascape genetics

We evaluated the proportional importance of geographical location (SPACE), sampling year (TIME) and water column dynamics (ENV) in explaining genetic connectivity patterns. To do so, the genotype matrix of each species was first converted into allele counts, where each row is an individual and each column indicates the count (0, 1 or 2) per allele. Redundancy analysis (RDA) identified the spatio‐temporal and environmental features explaining genetic (i.e. allelic) variation among the individuals of each species. RDA is a canonical extension of principal component analysis (PCA) in which the principal components produced are constrained to linear combinations of a set of predictor variables (Legendre & Legendre, 2012). The significance of RDA models was determined using 1,000 random permutations with the vegan package v2.5.6 in R (Oksanen et al., 2019). The RDA models were then subjected to forward selection using the ordiR2step function implemented in the vegan package, including a threshold of α = .05 and given the adjusted *R* ^2^ parameter of the RDA with all variables included to obtain an unbiased selection (Blanchet et al., 2008). Forward selection corrects for highly inflated type I errors and overestimated amounts of explained variation. The reduced set of explanatory variables based on forward selection was then used to recalculate the explained proportion of genetic variation.

Geographical variables (SPACE) were represented by Moran's Eigenvector Maps (MEMs), along with longitude and latitude. The MEMs were calculated for each individual species, based on a distance based matrix of the shortest geographical waterway distance between sampling locations (Table S1; Borcard & Legendre, 2002). Temporal variables (TIME) were represented by dummy variables from sampling year indicators. The year a sample was obtained was scored with the value 1; other years were marked with the value 0 (Table S1). Lastly, water column variables (ENV) for the greater North Sea area (including English Channel and Skagerrak) were downloaded from the ICES WGOOFE website (groupsites.ices.dk/sites/wgoofe) (Table S1). Apart from the commonly tested abiotic parameters in seascape genetic analysis such as sea surface and sea bottom temperature (SST and SBT, respectively, °C), and salinity of the surface and bottom waters (SSS and SBS, respectively, psu), also bottom dissolved oxygen concentration (O_2_, ml/L) and net primary production (PP; expressed as g C m^−2^ day^−1^) were included. Primary production was included as proxy for food availability for early life stages. Hydrodynamic parameters are included as our main aim was to investigate the potential effect of the Frisian Front in the Central North Sea. Frontal influences are represented by the depth of pycnocline (PYC, m) and a density‐based stratification index (STRAT, kg m^−3^ m^−1^) which describe the seasonal changes of the water column dynamics. Bottom shear stress (BSS, N/m^2^) represents the shearing force by the current. A detailed description of the water column dynamic parameters is available in the Supporting information Methods.

For each of the nine water column parameters, the monthly and yearly average were available for the period 1980–2004. The yearly standard deviation of each variable across the 12 months was calculated to capture the intra‐annual variation. Seascape genetic analyses were conducted with two sets of environmental variables (ENV1 and ENV2, Table S1). The first set (ENV1) consisted of 18 variables including the yearly average and standard deviation of each of the nine parameters. This set covers the broad environmental variation and captures the relevant biological information for a comprehensive analysis across the three species in an identical dynamic system. For the second set (ENV2), we selected specific month averages for each species. Specifically, we selected April, May, June and September for turbot (4 months; 36 variables), March, May, June and September for brill (4 months; 36 variables) and February, April and September for sole (3 months; 27 variables). This set allowed us to test whether specific seasonal variation in reproduction time (start and peak spawning time) affects the genetic variation among individuals. September was included as this month coincides with gonad development in adults and may have an effect of larval survivability of each individual species (Gibson et al., 2015).

Correlation plots (Figures S2–S7) and a classical principal component analysis (PCA; Figures S8‐S10) were used to study the correlation structure of the ENV1 and ENV2 variables. The results were inspected to verify the known environmental contrasts between marine regions. Furthermore, because correlated variables may interfere with each other during the forward selection procedure following RDA, Pearson correlations (*r*) > .8 were inspected to identify strong correlations between selected and nonselected environmental variables (see Supporting information Methods).

It should be mentioned that sole was included in the RDA analyses for comparison with the other species; however, caution is warranted when interpreting this comparison. In turbot and brill, almost every individual corresponds to an unique sampling location and therefore coincides with unique spatial and environmental parameter values. In contrast, the 720 genotypes of sole were obtained from only nine locations, and thus, the observations are associated with only nine unique spatial and environmental data points. As a result, the information content in the sole data is lower than for the two other species, that is fewer dimensions are available to attribute the genetic variation to spatial and environmental predictors. This different sampling design does not represent an inherent problem to estimate the variance components (see e.g. Raeymaekers et al., 2017), but the inflated degrees of freedom (720 instead of nine) make the associated P‐values less reliable.

## 3 RESULTS

### 3.1 Genetic diversity

No systematic linkage disequilibrium between loci pairs was detected across samples (Table S2). There was also no systematic locus‐specific deviation from Hardy–Weinberg equilibrium across samples. Estimates of expected heterozygosity of the samples varied between 0.621 and 0.680 in turbot, 0.743 and 0.781 in brill and 0.735 and 0.771 in sole (Table 2). The lowest heterozygosity of turbot was observed in the North Sea, while the highest values were found in the Bay of Biscay (Table 2). The lowest heterozygosity of brill was observed in the North Sea, while the highest values were found off Ireland. Most variation in heterozygosity levels was observed in sole, with samples from the Baltic Transition Region showing the lowest diversity. Heterozygote deficiency (i.e. *F*_IS_ > 0) was significant in 6 out of 23 samples for turbot, in 13 out of 23 samples for brill and in 5 out of 13 samples for sole (Table 2). Average allelic richness (AR) ranged from 5.71 in turbot and 7.11 in brill to 8.83 in sole. Turbot displayed the highest AR in the English Channel (AR = 5.91), whereas in brill and sole the highest estimates were observed in the German Bight (ENS09; AR = 7.57) and Irish Sea (IRS08; AR = 9.18), respectively (Table 2).

### 3.2 Population genetics

**FIGURE 2:**
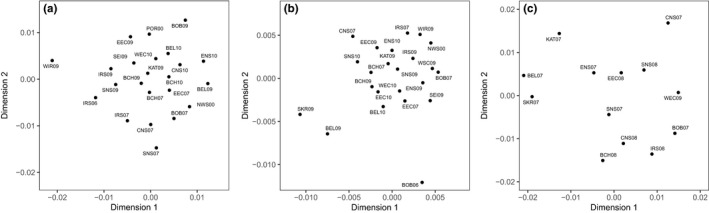
Nonmetric multidimensional scaling (NMDS) plots of three flatfish species based on pairwise *F*_ST_ values among samples. (a) Turbot (stress value: 0.22), (b) brill (stress value: 0.23) and (c) sole (stress value: 0.16). Information on the sample codes is available in Table 2

**FIGURE 3:**
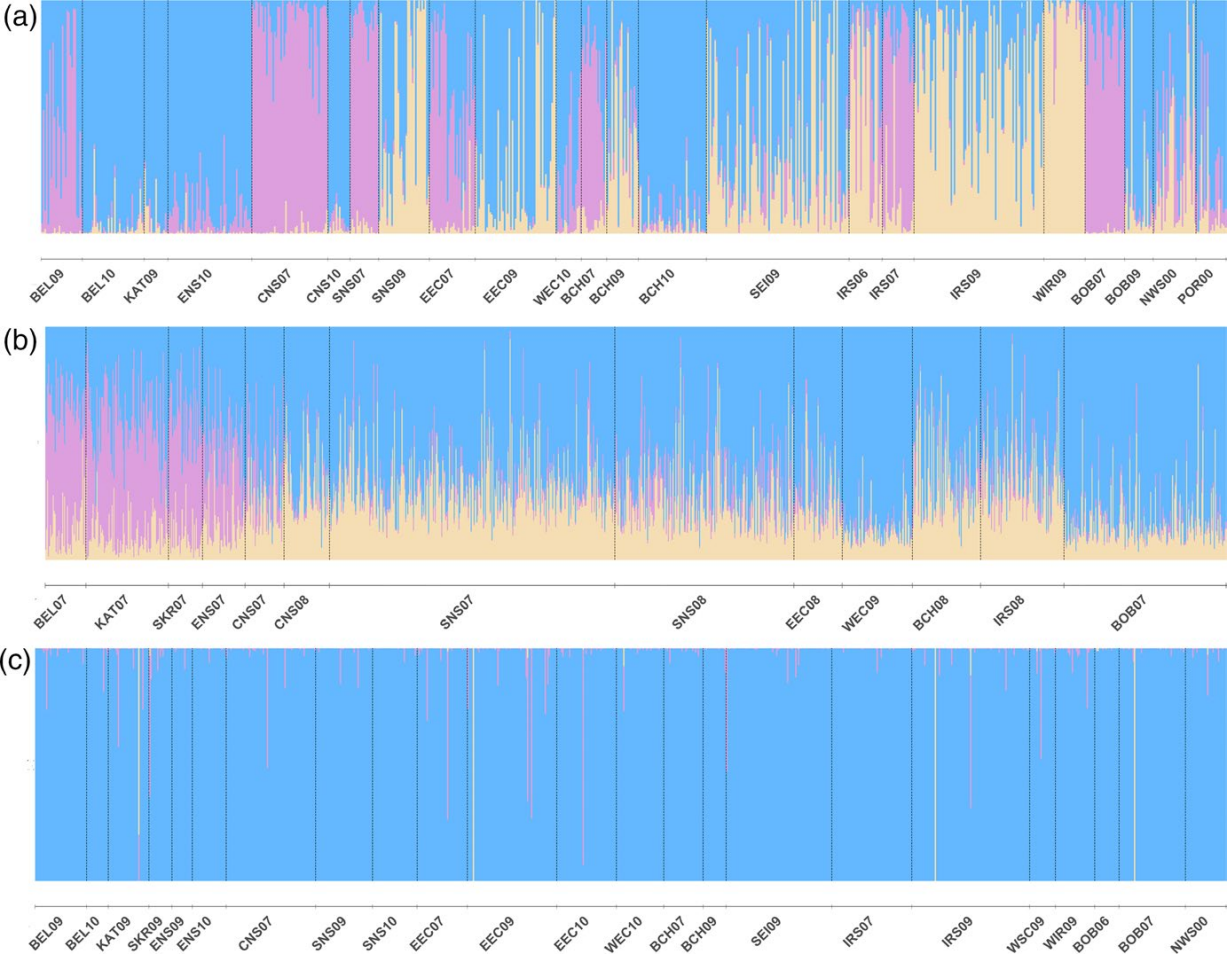
Individual assignment based on Bayesian clustering in STRUCTURE. Each bar represents an individual with its probability of membership to one of the hypothetical clusters. Cluster membership was estimated for (a) turbot with *K* = 3 clusters, (b) for sole with *K* = 3 clusters, and (c) for brill with *K* = 1 cluster. Information on the sample codes is available in Table 2

Overall genetic differentiation was similar among the three species: turbot (*F*_ST_ = 0.005; 95% CI = 0.003–0.008), brill (*F*_ST_ = 0.002; 95% CI 0.000–0.003) and sole (*F*_ST_ = 0.003; 95% CI = 0.001–0.004). Pairwise estimates of population differentiation ranged from zero to a significant maximum of 0.027 in turbot (WIR‐ENS, Table S3) and 0.012 in sole (SKR‐CNS, Table S5). No significant values were observed in brill after correcting for multiple testing, but the highest value was estimated between Skagerrak (SKR09) and the Bay of Biscay (BOB06) (*F*_ST_ = 0.016, Table S4). The NMDS plot of turbot pointed to a large cluster of almost all Atlantic samples, except for the West Coast of Ireland (WIR09) (Figure 2). The NMDS plot of brill revealed a split between the Baltic Transition Zone (BEL09 and SKR09) and a large Atlantic group, which included the 2010 Belt Sea (BEL10 and KAT09) samples. The Bay of Biscay (BOB06) clustered separately. No strong clustering was apparent for sole, but the two NMDS dimensions nevertheless separated the Baltic Transition Zone (KAT07, SK07 and BEL07), the North Sea (ENS07, CNS07, SNS07, SNS08 and EEC08) and an Atlantic group (BCH08, CNS08, WEC09, BOB07 and IRS08) (Figure 2). Bayesian clustering analysis pointed to four genetic clusters (*K* = 4) for turbot (Figure 3 and Figure S1). However, we opted to present three clusters that were relatively well segregated in space, which was not the case for any additional clusters at K = 4. One cluster predominantly covered the Baltic transition zone samples from Skagerrak (BEL), Kattegat (KAT) and German Bight (ENS). Interestingly, individuals belonging to the northern cluster occurred across the distribution range, but were almost absent from the southern and central North Sea (SNS). A second cluster consisted primarily of samples caught off the West coast of Ireland (WIR), but included individuals belonging to the northern/central North Sea (CNS). Individuals belonging to the third cluster were predominantly caught in the rest of the Atlantic Ocean (SNS, EEC, WEC, BCH, SEI and IRS) (Figure 3 and Figure S1). Similar to turbot, three genetic clusters (*K* = 3) are observed for sole. One cluster covers Skagerrak, Kattegat and German Bight (BEL, KAT, SKR and ENS). A second cluster occurred throughout the distribution range of sole, but was more pronounced in the Bay of Biscay (BOB). The third cluster had a wider distribution across the Eastern English Channel, Irish Sea and North Sea. In contrast to turbot and sole, Bayesian clustering did not subdivide brill into genetically distinct subgroups (*K *= 1, Figure 3 and Figure S1). Subsequent seascape analyses focused on subtle genetic patterns within the single population of brill and genetic differentiation between populations of turbot and sole.

### 3.3 Seascape genetics

The genetic variation among individuals of each species was partitioned with RDA into variation attributable to geographical location (SPACE), water column variables (ENV) and the effect of sampling across different years (TIME) (Table 3). We first considered the RDA based on ENV1 (i.e. the subset of nine yearly averages and nine standard deviations). Overall, RDA explained more variation in turbot (ENV + SPACE+TIME; *R* ^2^adj = .018), followed by brill (*R* ^2^adj = .010) and sole (*R* ^2^adj = .001). In turbot, SPACE accounted for the largest significant single fraction of explained variation (*R* ^2^adj = .013). The effect of TIME (*R* ^2^adj = .003) was also significant, and both effects remained after control for ENV (SPACE|ENV: *R* ^2^adj = .014; TIME|ENV: *R* ^2^adj = .003). SPACE also accounted for the largest significant single fraction of explained variation in brill (*R* ^2^adj = .007). The effect of ENV was also significant (*R* ^2^adj = .004), and both effects remained after control for TIME (SPACE|TIME: *R* ^2^adj = .007; ENV|TIME: *R* ^2^adj = .004). In sole, the only significant single fraction of explained variation was TIME (*R* ^2^adj = .001). This effect remained after control for ENV (TIME|ENV: *R* ^2^adj = .001).

**TABLE 3:** Partitioning of genetic variation among individuals of turbot, brill and sole. Analyses were conducted for the greater North Sea area, including the Eastern English Channel and the Baltic Transition Zone. Results are based on 18 environmental variables, i.e. the yearly average and standard deviation of each of the nine focal parameters (see Materials and Methods). Adjusted variance components (*R* ^2^adj) quantify the full or partial fractions explained by environment (ENV), space (SPACE) and time (TIME). Significant *p*‐values (<.05) for these fractions of variation are in bold. Co‐variables significantly associated with genetic variation after forward selection are reported (PYC_SD = standard deviation of the depth of the pycnocline and STRAT = stratification index)

Turbot					Brill			Sole	
		*R* ^2^adj	*p*‐value		*R* ^2^adj	*p*‐value		*R* ^2^adj	*p*‐value
*N*	238			359			720		
Total variation	4,633.8			8,193.1			11,569		
ENV		.004	.155		.004	**.043**		.002	.064
SPACE		.013	**.020**		.007	**.043**		.002	.058
TIME		.003	**.012**		.000	.611		.001	**.029**
ENV + SPACE		.018	**.010**		.010	**.017**		.001	.212
ENV + TIME		.007	**.043**		.003	.060		.002	**.017**
SPACE + TIME		.013	**.030**		.007	.059		.002	**.025**
ENV + SPACE + TIME		.018	**.014**		.010	**.019**		.001	.162
ENV|SPACE + TIME		.005	.149		.003	.127		.000	.772
SPACE|ENV + TIME		.011	.076		.006	.060		.000	.855
TIME|ENV + SPACE		.000	.346		.000	.851		.000	.264
ENV|TIME		.004	.137		.004	**.034**		.002	.057
ENV|SPACE		.005	.178		.004	.088		.000	.724
SPACE|TIME		.010	.068		.007	**.041**		.001	.067
SPACE|ENV		.014	**.020**		.007	.064		.000	.677
TIME|ENV		.003	**.015**		.000	.638		.001	**.032**
TIME|SPACE		.000	.529		.000	.568		.004	.101
Residuals		.982			.990			.999	
Forward selection									
ENV									
	PYC_SD	.002	**.014**	STRAT	.002	**.008**			
SPACE									
	MEM27	.003	**.002**	MEM46	.001	**.006**			
	MEM8	.006	**.008**	MEM2	.003	**.008**			
	MEM28	.008	**.030**	MEM4	.004	**.012**			
	MEM13	.010	**.030**	MEM45	.005	**.012**			
	MEM1	.011	**.042**	MEM31	.006	**.016**			
TIME							2007	.001	**.02**

In a second step, forward selection was applied to reduce the predictor variables to only those variables that significantly correlated with the observed genetic variation among individuals. In turbot, this analysis revealed a significant effect of the variation in the depth of the pycnocline (PYC_SD), in addition to effects of five species‐specific MEMs (MEM27, MEM8, MEM28, MEM13 and MEM1; Table 3). In brill, forward selection revealed a significant association with the average stratification index (STRAT), also in addition to effects of five species‐specific MEMs (MEM46, MEM2, MEM4, MEM45 and MEM31; Table 3). In sole, the genetic variation among individuals was only associated with sampling year 2007.

RDA based on the same spatial and temporal matrix, but including specific biologically relevant environmental monthly averages for each species (i.e. ENV2), provided quantitatively similar results to the previous analysis (Table S6). In turbot, forward selection revealed significant effects of the sea surface temperature and sea bottom salinity in September (SST_Sept and SBS_Sept; Table S6). In brill, we observed a significant effect of the stratification index in June (STRAT_Jun; Table S6). When recalculating the explained proportion of genetic variation after forward selection, SPACE remained more important than ENV in both turbot and brill (Table S7). The effect of ENV remained significant after control for SPACE in turbot, but became nonsignificant in brill (Table S7). In sole, only TIME remained significant, so this analysis was not performed.

## 4 DISCUSSION

Our comparative interspecific seascape analysis reveals unique and shared characteristics among three codistributed and commercially exploited flatfishes in the North East Atlantic region. While focusing on the same geographical area and using the same type and comparable number of molecular markers, we found no genetic structure in brill and confirmed the weak genetic differentiation of sole and turbot with traditional population genetic analyses. Seascape analysis pointed to the contribution of environmental factors such as stratification (brill) and the variation in pycnocline depth (turbot) to the genetic patterns. These variables highlight the impact of hydrodynamic features on gene flow in two of three investigated flatfishes. Below, we discuss the subtle genetic structure and seascape of each of the three species and explore the integrative management of their fishery.

### 4.1 Genetic structure

Our observations on spatial differentiation are in agreement with previous population genetic studies. We confirm that sole populations group in a Baltic, northern and southern Atlantic population, the Atlantic populations showing a pattern of isolation by distance (Cuveliers et al., 2012; Diopere et al., 2018). Turbot populations of the Baltic Sea are clearly differentiated from the North East Atlantic Ocean; they co‐occur in a hybridization zone between the North Sea and Holocene Baltic Sea. The North East Atlantic Ocean range of turbot is structured in a western Irish shelf and northern North Sea population and an Atlantic population (Le Moan et al., 2019; Prado, et al., 2018; Vandamme et al., 2014). We confirm the allozyme‐based finding of Blanquer et al. (1992) that brill represents a spatially homogenous population across its Atlantic range. Seascape genetic analysis suggests, however, that more subtle forces may differentiate populations within each of these species.

The genetic structure or the lack thereof in these three commercial flatfishes in the North East Atlantic region fits with the current understanding of marine population dynamics. Large census and effective population sizes characterize sole populations despite a historically high fishing pressure (Cuveliers et al., 2011; ICES, 2020). In contrast, census population sizes of turbot and brill are an order of magnitude smaller. The lower genetic diversity of turbot than other fishes might be indicative of a smaller effective population size and/or above average fishing pressure (DeWoody & Avise, 2000; Pinsky & Palumbi, 2014). Dispersal potential of all three flatfishes is high during the larval stage (Barbut et al., 2019; Bolle et al., 2009; Lacroix et al., 2013) and adult stage (Burt & Milner, 2008; van der Hammen et al., 2013; Lecomte et al., 2020), but low during the juvenile stage. Unlike adults who make spawning and feeding migrations, planktonic larval drift is under subtle biological control and first year flatfish grow out in shallow water coastal nursery grounds (Le Pape & Cognez, 2016). Hydrodynamic variables pycnocline and stratification were included in the analysis to investigate whether hydrodynamic fronts may have an effect on the dispersal potential of larvae phases and subsequently affect genetic differentiation. Dispersal of all three species is influenced by salinity gradients, either as a barrier to estuarine and brackish ecosystems (sole and brill) or as an adaptation to brackish ecosystems (turbot lives in the Baltic Sea) (Nielsen et al., 2004). The offshore and active lifestyle of (sub)adult turbot and brill increases the chances to encounter a diversity of water masses and oceanographic barriers, such as stratification fronts (Barbut et al., 2019; Vandamme et al., 2014). Sole populations live in well‐mixed coastal regions, often associated with macrobenthic communities at the mouth of estuaries, inducing coastal differentiation (Darnaude et al., 2004; Darnaude et al., 2004).

### 4.2 The seascape of flatfish

In this study, fluctuating environments likely influence the dispersal potential and hence the genetic structure of flatfishes sharing the shelf habitat and belonging to the same community. In turbot, genetic variation was best explained by sampling location (SPACE), followed by sampling year (TIME). The global contribution of water column variables (ENV) was weak, but a few key environmental variables associated with genetic variability emerged via forward selection: the variability (*SD*) in the depth of the pycnocline, and the sea surface temperature and sea bottom salinity in September. In a data set of genotypes extended with the Baltic Sea, Vandamme et al. (2014) additionally identified in turbot the effects of bottom shear stress and dissolved O_2_, all factors pointing to the influence of the water column. Genetic variation of brill was best explained by sampling location (SPACE), followed by water column variables (ENV). A single key environmental variable, stratification, was identified based on forward selection. The identification of spatial and environmental determinants of genetic structure in this species based on RDA is remarkable given its extremely shallow population structure revealed by other methods. There was no contribution of sampling location (SPACE) or water column variables to the genetic variation of sole. Instead, genetic variation was attributed to variation between years (TIME). A previous study in sole highlighted winter sea temperature, food availability and coastal currents as the major determinants of genetic structure (Diopere et al., 2018). This study differs from the current one in that the RDA was performed based on more than 400 SNPs, rather than 10 microsatellite markers. Hence, an underestimate of the effect of SPACE and ENV may apply to the current study due to differences in the type or number of genetic markers used. However, Diopere et al. (2018) also included samples across a larger geographical range, and thus, the studies are not fully comparable.

Overall, the RDAs in our study provided limited explanation of the total variance, a feature common to studies based on a small set of molecular markers (Gagnaire, 2020). Multimarker studies tend to have more power to attribute variance to the environment (Benestan et al., 2016; Coscia et al., 2020). Yet, an additional major difference between studies concerns the use of population‐based versus individual‐based analyses to identify the main spatial, environmental or temporal determinants of population structure. In this study, the RDAs were performed in turbot and brill at the individual level to make optimal use of the available spatial and environmental information and to allow direct comparison between the three species. No dimension reduction of the genetic response matrix was applied for this study, and hence, the low adjusted *R* ^2^ values correctly reflect that only a fraction of the large genetic variation among individuals is attributed to external factors.

Our aim to correlate the population genotypes of three flatfishes with environmental factors, and hence to contribute knowledge on their lifestyles, is reflected in the selection of the parameters and variables. We opted for the yearly average and standard deviation of each of the nine parameters in RDA analysis ENV1 to cover the broad environmental variation and to capture the relevant biological information across all three species. This approach turned out to explain as much of the variance as a second set (ENV2), where we opted for species‐specific seasonal variation in reproduction time (start and peak spawning time). Spring and early summer are of crucial importance for the connectivity and survival of the larval stage of all three species across the northern range of their distribution. September was chosen in all three species because of the importance of maternal effects on gonad development in adults and hence egg quality in fall (Gibson et al., 2015).

Modulation of dispersal due to the interaction of environment and species‐specific life‐history traits is an important factor impacting the subtle genetic patterns. Flatfish disperse during two phases, the larval and (sub)adult stage, in the life cycle. Larvae are advected over considerable distances during the planktonic phase (Gibson et al., 2015). The timing and location of spawning and the development of the larvae shape the drift pattern (Barbut et al., 2019; Lacroix et al., 2013). Drift time is related to planktonic larval duration and hence inversely related to growth rate and temperature (Shanks, 2009). Sole spawns during the cooler spring, larvae grow slower and drift longer than turbot (140 km versus 102 km on average) (Barbut et al., 2019). Turbot spawns on average later in the season than brill and sole, at a time when average temperatures are higher and oceanographic barriers more pronounced. Its larvae drift over shorter distances and hence may have a higher survival rate than brill and sole (Table 1). The shorter modelled drift distance of larval turbot implies a higher potential for spatial differentiation than sole and brill (Table 1). The changes in water column density in fall (pycnocline depth, and temperature and salinity in September) coincide with the fall breakdown of the pycnocline and stratification and match with the end of larval settlement in the coastal nurseries. Our initial hypothesis that September is an important month for gonad maturation might be complemented with the end of larval dispersal and settlement. Strong shifts in the stratification index and pycnocline are associated with seasonal fronts in the tidally mixed North Sea and English Channel (van Leeuwen et al., 2015; Vandamme et al., 2014). The summer‐spawned larvae of turbot might experience either northern stratified or southern well‐mixed waters, separated by seasonal fronts across the North Sea. Oceanographic barriers are common causes of spatial patterning (Galarza et al., 2009; Miller et al., 2015). Interestingly, dispersal modelling shows a link between the larval life history and hydrodynamics; stratification shapes the drift pattern of turbot larvae with high self‐recruitment and some exchange from North to South in the North Sea throughout summer (Barbut et al., 2019).

The influence of yearly averaged stratification and fall water mass (September temperature and salinity) on brill is difficult to explain. Unlike turbot, brill spawns over a longer period but similar to turbot and sole settles in coastal nurseries. The spring‐spawned larvae experience a tidally and wind‐mixed environment. The homogeneous genetic structure does not match with the modelled larval dispersal, where the North Sea populations are characterized by a high level of self‐recruitment (Barbut et al., 2019). We hypothesize that the low spawning density and undocumented (sub)adult behaviour of brill are important factors in addition to larval dispersal.

In our study, none of the environmental parameters correlated with genetic variation in sole, which we attribute to the geographically restricted sampling and the limited number of markers compared to Diopere et al. (2018). The pattern of isolation by distance of the Atlantic population has been attributed to a coastal lifestyle, including the associated susceptibility to winter mortality and year‐through mixing of the water column (Darnaude, et al., 2004; Diopere et al., 2018; Rijnsdorp et al., 1992). Biophysical modelling attributes considerable larval connectivity to sole across distances in the range of 100 km (Barbut et al., 2019; Lacroix et al., 2013). Its genetic structure, life history, dispersal dynamics and general biology differentiate sole clearly from the congeneric turbot and brill. Overall, we cannot ignore the possibility that the observed disparity between genetic structure and modelled larvae dispersal is due to the lack of power of the microsatellites used to detect subtle levels of genetic differentiation.

Early life dispersal dynamics are complemented with adult‐mediated population connectivity (Frisk et al., 2014; Huijbers et al., 2013). (Sub)adults contribute considerably to dispersal (Hunter et al., 2003). While fish egg and larval advection are under subtle biological control of nycthemeral migration and retention strategies, adults may actively move between winter and summer grounds and between feeding and spawning grounds (Gibson et al., 2015; Harden Jones, 1968; Hunter et al., 2003). Some taxa such as clupeids, salmonids and tunas make long‐distance annual migrations. Hence, local population size and structure may be strongly influenced by emigration and immigration of individuals. Adult‐mediated population connectivity (AMPC) in addition to larval dispersal leads to a greater scale of interpopulation movement than solely anticipated from larval dispersal (Frisk et al., 2014). Site fidelity may be strong in flatfish (Dando, 2011; Hunter et al., 2003). Sole disperses essentially at night over distances of less than 100 km in the northern range of the Atlantic Ocean (Lecomte et al., 2020). Seasonal movement between the feeding and spawning grounds is the rule (Burt & Milner, 2008). Although turbot has a large capacity to disperse with a distinct diurnal rhythm and greater activity at night (Lagardère et al., 1995), dispersal of juveniles turned out to be limited (Prado, et al., 2018). Strong fidelity to the spawning sites and limited dispersal of adults during the reproduction period have been documented in the Baltic Sea (Florin & Franzen, 2010), suggesting geographical segregation. Brill has a capacity similar to turbot to disperse, but scientific evidence is lacking. Other flatfish such as European plaice (Hunter et al., 2003; Ulrich et al., 2017) and Pacific halibut (Gibson et al., 2015) disperse seasonally over long distances, but retain distinct population patterns. In general, the scale of differentiation of many species is smaller than their dispersal capacity (Frisk et al., 2014; Mullon et al., 2002). Hence, AMPC may maintain either a high stock connectivity or enhance local stock structuring (Frisk et al., 2014). A consequence is that individual‐based models of dispersal should include the full life cycle, in addition to life stage‐specific behavioural and metabolic dynamics (Teal et al., 2012). Our study confirms the weak neutral population structure often found in marine organisms because of the high level of connectivity and the large size of the populations (Hauser & Carvalho, 2008). Regardless, a comparative seascape genetic analysis illustrated the potential to detect subtle signals of differentiation due to the interaction between environmental dynamics and early life stage dispersal.

### 4.3 Integrative management of the flatfish fishery

Ecosystem‐based fisheries management (EBFM) considers population genetic insights an important aspect of biodiversity conservation (Casey et al., 2016). EBFM also acknowledges that multispecies comparisons are an important step in the process towards a holistic management of natural resources. The functioning of communities depends on close interaction between populations and species (van der Plas, 2019).

The genetic structure investigated in this study compares three similar flatfishes. Whereas sole is biologically well‐documented, limited information is available for the closely related species turbot and brill. Interestingly, population structure of these three species showed differences, even when using neutral microsatellites. The phylogenetically closely related turbot and brill display similar historical expansion and recolonization patterns of the North East Atlantic Ocean and greater similarities in life‐history traits than sole (Table 1; Vandamme, 2014). Regardless, the level of genetic differentiation and heterozygosity is most differentiated between turbot and brill, suggesting the influence of local environmental features and species‐specific traits on genetic patterns.

The strength of this study is the comparative approach, which confirms the role of subtle physical barriers on species‐specific traits and subsequently on differentiation. For example, year‐to‐year variability in advection determines recruitment, and hence cohort structure and composition (Guinand et al., 2013; Hauser & Carvalho, 2008; Hjort, 1914) and community structure (Selkoe et al., 2016). The life‐history traits of the spring spawners brill and sole and summer spawner turbot may impact genetic differentiation. Hydrodynamic fronts correlate with a north–south stock division of turbot in the North Sea. This is also the case for impact on the spawning locations of fish (Sinclair & Iles, 1985) and the nonrandom dispersal of fish larvae (Galarza et al., 2009; Galindo et al., 2010). In addition, the physical characteristics of the water mass shape the heritable behaviour of marine organisms. This is illustrated by fjord and offshore Atlantic cod mixing in the fjords of the Skagerrak and Kattegat area, but only inshore cod spawns in the fjords. The latter has a chromosomal rearrangement critical for larval survival at low salinities (Barth et al., 2017). In another case, the genetic structure of American lobster along the East Coast of Canada is mediated at three genes by the regional currents and thermal adaptation (Benestan et al., 2016).

European fisheries management advice provided by ICES is based on single species stock assessments. For all three species, ICES provides catch advice for the North Sea stock, which includes the English Channel for brill (ICES, 2020). The Baltic Transition Area is considered a separate stock (ICES, 2020). While the subdivision between the Baltic Transition Area and North Sea would not be required for brill, the seascape and genetic analysis suggests a dispersal limitation within the North Sea for all three species (at the level of the Frisian Front). Further inconsistencies between ICES catch advice and our genetic analysis are observed for turbot and sole. Our results illustrate a separate population of Western Irish Sea turbot, but no catch advice is provided for turbot outside the North Sea area. Taking into account that turbot seems less resilient genetically, with a below‐average genetic diversity (DeWoody & Avise, 2000) and the loss of the Turbot Bank spawning population (North Sea, off Scotland) (Kerby et al., 2013), catch advice for additional areas is recommended. At the same time, the fragmented catch advice for sole (North Sea, Eastern English Channel, Western English Channel, and so on) is unwarranted based on the current population genetic analysis and additional studies by Diopere et al. (2018) using outlier loci.

Based on the current results, it is too early to make recommendations for an EBFM approach for North Sea flatfish. The small number of neutral microsatellite loci does not have sufficient power to unequivocally resolve the genetic populations pattern. Further research is recommended to advise a sound EBFM. The use of markers susceptible to adaptive processes has proven to detect fine‐scale local differentiation in both turbot (Vandamme et al., 2014) and sole (Diopere et al., 2018). Several studies in the marine environment illustrated that oceanographic barriers are a common cause of spatial patterning (Galarza et al., 2009; Miller et al., 2015). The increasing maturity of biophysical dispersal models, including those targeting flatfishes (Barbut et al., 2019; Jonsson et al., 2016), provides unique opportunities to model gene flow and adaptation, and hence recruitment. Lacroix et al. (2013) predicted recruitment variability of one‐year‐old sole with a biophysical dispersal model. Hence, biophysical models, in addition to population dynamic models, will increasingly provide input for stock assessment (Baltazar‐Soares et al., 2018; Miller, 2007). Regional models will facilitate multispecies management because they use the same hydrodynamic model adapted with population‐specific parameters of biological traits.

In summary, the distinct genetic patterns of three codistributed and commercially exploited flatfishes are linked with their environment, habitat preferences and life history. The well‐documented genome of turbot and the closely related Senegal sole (Louro et al., 2020; Prado, et al., 2018) open perspectives for understanding adaptation in a high gene flow context. In a further time frame, a comprehensive population‐specific full life cycle model in support of fisheries management would benefit from the integration of the dispersal dynamics of the planktonic and adult phase of flatfish.

## 5 DATA ARCHIVING STATEMENT

All data and codes used in this article are publicly available on Dryad (https://doi.org/10.5061/dryad.h70rxwdgq).

## CONFLICT OF INTEREST

None declared.

## Supporting information

Supplementary Material
