# Supplementary material for: Reconciling seascape genetics and fisheries science in three codistributed flatfishes
Source: Evol Appl. 2020 Nov 2;14(2):536–52. doi: 10.1111/eva.13139 (PMC7896710; doi:10.1111/eva.13139)
Supplement: Supplementary file 1 — Supplementary Material [file EVA-14-536-s001.docx]

**Supplementary material**

**Materials and methods**

***Seascape genetics***

The environmental variables reflecting the water-column dynamics were downloaded from the ICES WGOOFE website. Each individual sample was linked to the environmental data by extracting the value of the closest cell without extrapolation. As the raw environmental model available from the ICES-WGOOFE database has a fine-grained geographical scale, each sampling location was assigned a unique value for each of the environmental variables.

In total nine environmental parameters, of which six abiotic parameters and three hydrodynamic parameters, were analysed (Table S2). Those variables were chosen that influence the distribution of marine species and vary between sampling locations. Some parameters (e.g. temperature) vary more than others (e.g. salinity), and some differences become more pronounced during specific seasons. For other parameters, annual estimates of environmental data may differ substantially because of seasonal effects (e.g. depth of pycnocline). Therefore, monthly (12 months x 9 parameters) and yearly (1 x 9 parameters) averages were calculated over the available period 1980-2004. In addition, the yearly standard variation of each parameters over the same time period (1 x 9 parameters) was included to test whether large seasonal variation of a specific variable might affect the level of genetic variation of each species differentially, leaving a total of 126 variables to be tested.

Two hydrodynamic variables are included to investigate the potential effect of hydrodynamic fronts in the Central North Sea. These hydrodynamic barriers are represented here by the depth of pycnocline (PYC, m) and a density-based stratification index (STRAT, kg.m^-3^.m^-1^). PYC represents the depth at which the combined effect of temperature and salinity changes over the year and results in an abrupt density change or pycnocline. STRAT gives the maximum value of this vertical gradient. In shallower water the stratification index will correspond with the depth of the water (so the entire water column is mixed), like in the southern North Sea. In deeper water the density begins to change more abruptly. Layering in the ocean is an important feature that influences biological, chemical and physical processes. Bottom shear stress (BSS, m^2^ s^-2^) represents the shearing force originated by the currents. This factor relates to the amount of diatoms and primary production that is buried in the sediment. Large differences can be observed over the study area, e.g. due to turbulent conditions in the southern North Sea some 1% of the annual primary production is buried in the sediment. On the other hand, the stable haline stratification of the Baltic Sea results in negligible sediment fluxes. In the German Bight a maximum integrated biomass is found due to the vertical transport driven by near bottom inflow of saltier North Sea water and a considerable river runoff. As the level of bottom shear stress is also affected by the sediment type, the effect of sediment type on an organism is indirectly included in the analysis. For more detailed information on these variables see Schrum, Siegismund, & John ([2003](#_ENREF_14)) and Vandamme et al. ([2014](#_ENREF_16)).

The seascape genetic analyses with RDA (partitioning the genetic variation in spatial (SPACE, environmental (ENV) and temporal (TEMP) factors) were conducted based on two reduced sets of environmental variables. One dataset (ENV1) included the yearly average and standard deviation of each of the nine parameters which allowed to use the same set of variables for all species. A second dataset (ENV2) comprised species specific monthly averages related to the start and peak spawning season of (see Table 1) and the monthly averages for September which is for all species related to larval survival and gonad development. Specifically, we selected April, May, June, and September for turbot (4 months; 36 variables), March, May, June, and September for brill (4 months; 36 variables), and February, April, and September for sole (3 months; 27 variables). SPACE was always included as Moran’s Eigenvector Maps (MEMs) based on waterway distances (see methods), and TIME was included as year dummy variables.

Correlation plots of the ENV1 and ENV2 data set used in the seascape genetic analysis with RDA are presented in Figure S2-S7. A classical PCA method was used to screen the major gradients present in the ENV1 datasets used for the seascape analysis with RDA (Fig. S8, S9 and S10). For turbot, PCA analysis showed that the first principal component (PC I) explained 67% of the variation, and was positively correlated with oxygen concentration, depth of pycnocline, standard deviation of temperature and salinity, stratification, and negatively correlated with primary production, temperature and salinity and bottom shear stress. PC II explained 15% of the variation, mainly representing bottom temperature and standard deviation of the depth of the pycnocline (Fig. S8). For brill, PC I and PC II explained 64% and 13% of the variation, respectively. Similarly to turbot, PC I was positively correlated with the standard deviation of temperature and salinity, stratification, oxygen and depth of the pycnocline, while PC II was positively correlated with standard deviation of the depth of the pycnocline (Fig. S9). For sole, PC I and PC II explained 60% and 16% of the variation, respectively. PC I was positively correlated with the standard deviation of temperature and salinity, stratification and depth of the pycnocline, while PC II was positively correlated with bottom salinity and negatively with oxygen (Fig. S10).

Because correlated variables may interfere with each other during the forward selection procedure following RDA, Pearson correlations (r) > 0.8 were inspected to identify strong correlations between selected and non-selected ENV1 and ENV2 variables. The resulting clusters of correlated variables are highlighted in the legend of Figure S2-S7. Given the association of the selected environmental variables with individual allele counts, these clusters may indicate which general oceanographic features potentially affect gene flow in each species.

The results of the RDA analysis based on ENV1 and ENV2 are depicted in Table 3 and Table S6, respectively. Parsimonious RDA models (i.e. based on variables identified with forward selection) are presented in Table S7.

**Supplementary tables**

**Table S1.** Overview of the variables included in the seascape analysis: Geographic variables (SPACE) were represented by Moran’s Eigenvector Maps (MEMs), along with longitude and latitude. Temporal variables (TIME) were represented by dummy variables (0 or 1) from sampling year indicators. Nine water-column variables (ENV) were included for the greater North Sea area, of which six abiotic parameters and three hydrodynamic parameters.

**Table S2.** Marker pairs showing significant linkage disequilibrium in three flatfishes. Linkage disequilibrium was tested using Fisher’s exact test in the GENEPOP package in R. Significance was determined after Bonferroni correction for multiple testing.

Table S3. Estimates of pairwise *F_ST_* for all 14 microsatellites of turbot (below diagonal). Significant *p*-values (< 0.05; above diagonal) are in bold and significant values after controlling for the false discovery rate (FDR) are marked with an asterisk.

Table S4. Estimates of pairwise *F_ST_* for all 14 microsatellites of brill (below diagonal). Significant *p*-values (< 0.05; above diagonal) are in bold and significant values after controlling for the false discovery rate (FDR) are marked with an asterisk.

Table S5. Estimates of pairwise *F_ST_* for all 10 microsatellites of sole (below diagonal). Significant *p*-values (< 0.05; above diagonal) are in bold and significant values after controlling for the false discovery rate (FDR) are marked with an asterisk.

**Table S6.** Partitioning of genetic variation among individuals of turbot, brill and sole. Analyses were conducted for the greater North Sea area, including the Eastern English Channel and the Baltic Transition Zone. Results are based on the second set of environmental variables, the species specific month averages. Adjusted variance components (R²adj) quantify the full or partial fractions explained by environment (ENV), space (SPACE) and time (TIME). Significant *p*-values (< 0.05) for these fractions of variation are in bold. Co-variables significantly associated with genetic variation after forward selection are reported (SST_Sept = Sea Surface Temperature in September, SBS_Sept = Sea Bottom Salinity in September, and STRAT_Jun = stratification index in June)

**Table S7.** Partitioning of genetic variation among individuals of turbot and brill with parsimonious variables (i.e. after forward selection). Analyses were conducted for the greater North Sea area, including the Eastern English Channel and the Baltic Transition Zone. Adjusted variance components (R²adj) quantify the full or partial fractions explained by environment (ENV) and space (SPACE). Significant *p*-values (< 0.05) for these fractions of variation are in bold. (A) Results based on the parsimonious variables from Table 3 (turbot: PYC_SD for ENV versus MEM27, MEM8, MEM28, MEM13 and MEM1 for SPACE; brill: Strat for ENV versus MEM46, MEM2, MEM4, MEM45 and MEM31 for SPACE). (B) Results based on the parsimonious variables from Table S6 (turbot: SST_Sept and SBS_Sept for ENV versus MEM27, MEM8, MEM28, MEM13 and MEM1 for SPACE; brill: Strat_Jun for ENV versus MEM46, MEM2, MEM4, MEM45 and MEM31 for SPACE).

**Supplementary figures**

1. Turbot


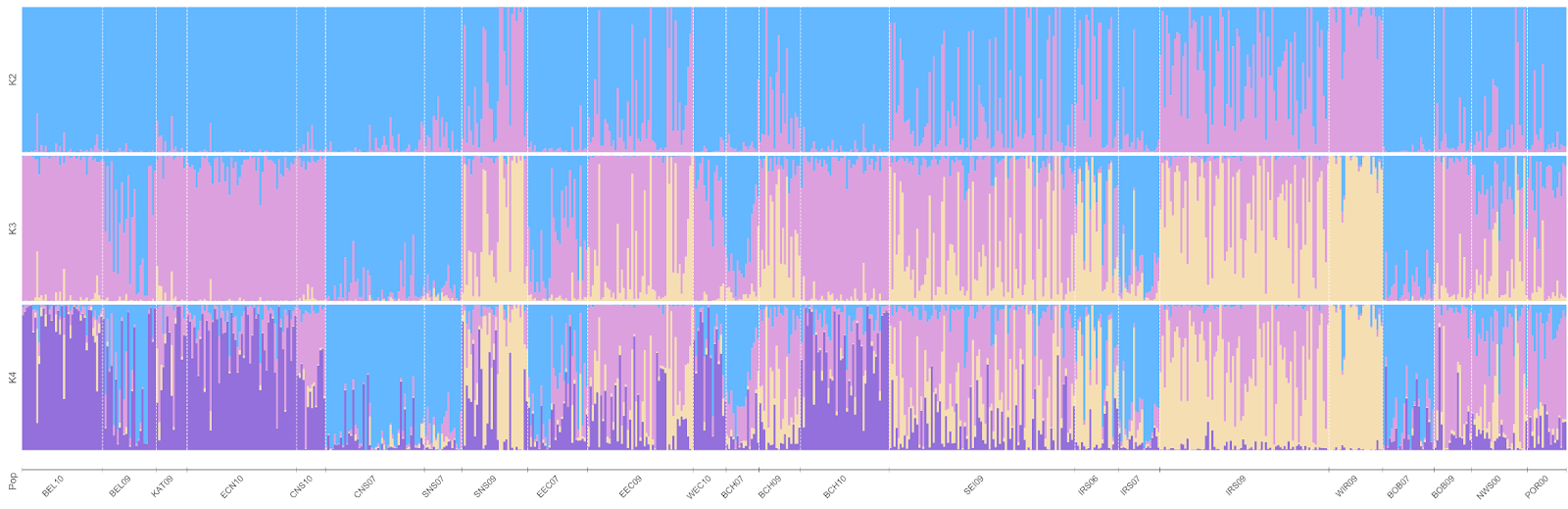


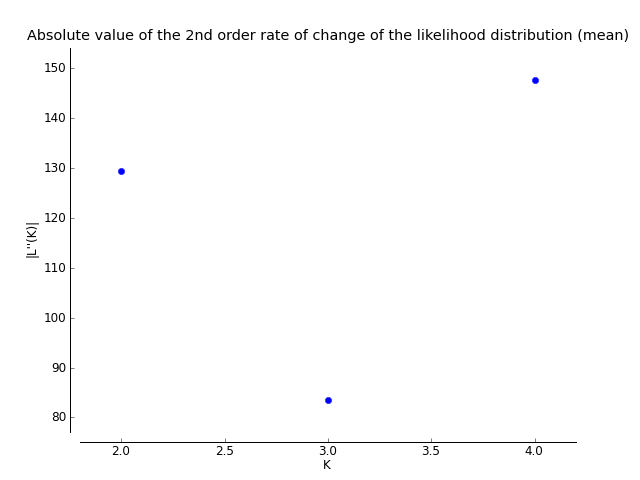


1. Brill


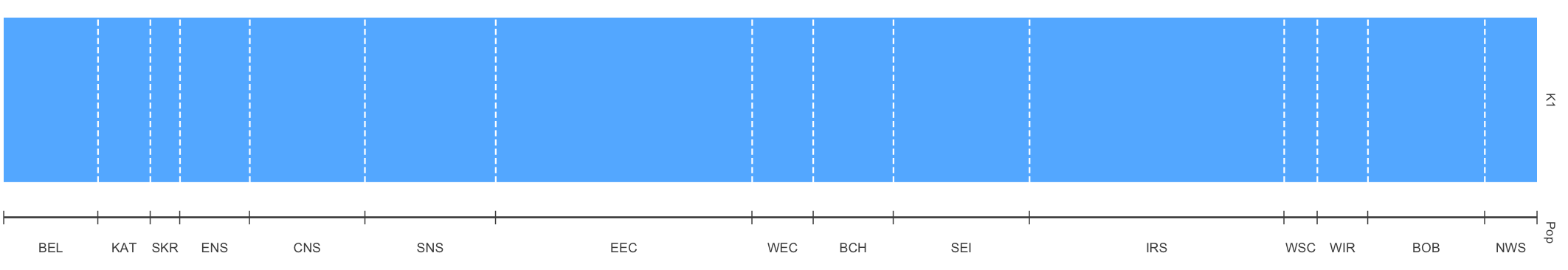


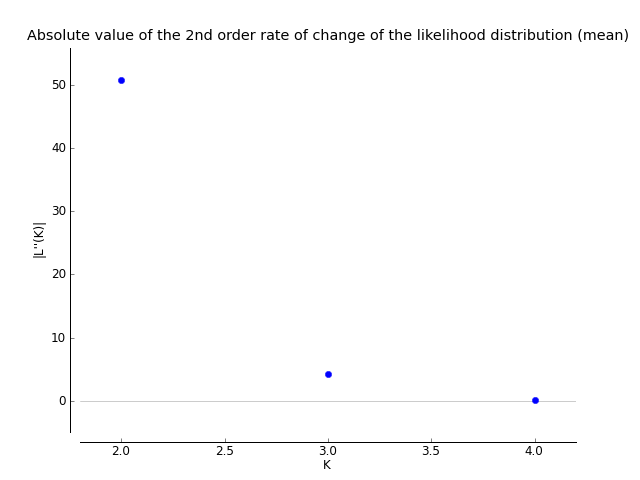


1. Sole


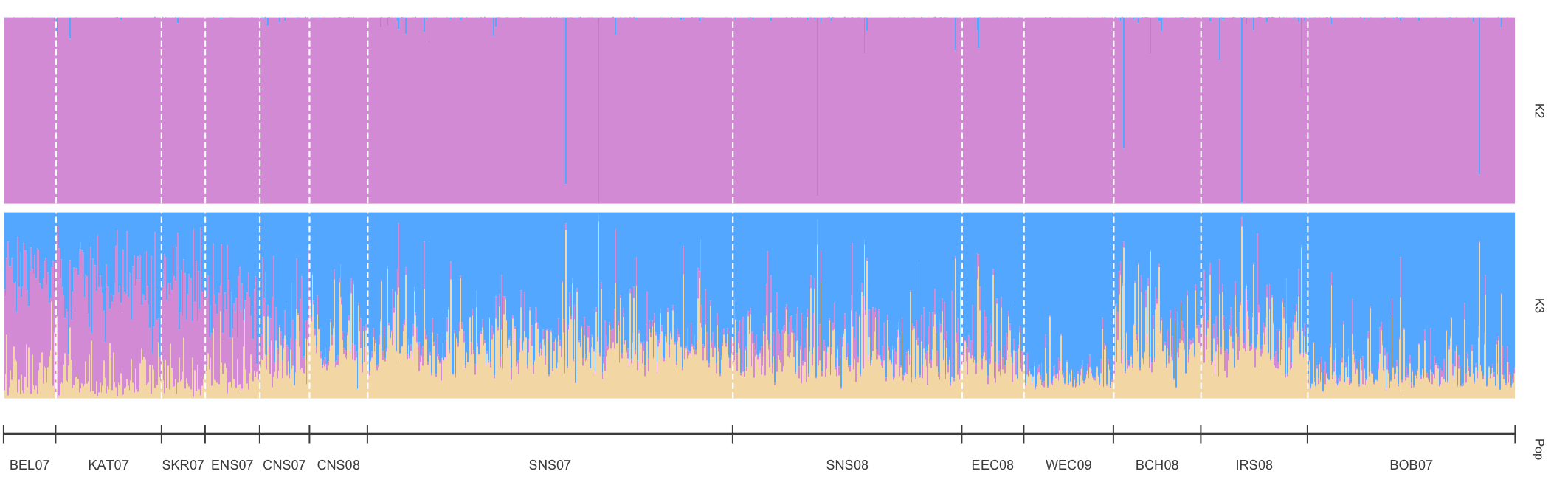


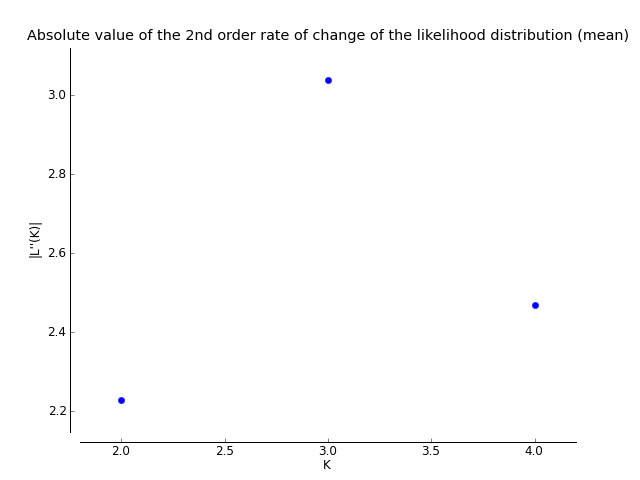


**Figure S1.** Individual assignment based on Bayesian clustering as implemented in STRUCTURE. Each bar represents an individual with its probability of membership to one of the hypothetical clusters. Cluster membership for (A) turbot genotyped at 14 microsatellite markers and 23 samples at K = 2, 3 and 4. (B) Cluster membership for brill genotyped at 14 microsatellite markers and 23 samples at K = 1. (C) Cluster membership for sole genotyped at 10 microsatellite markers and 13 samples at K = 2 and 3 . Plots of delta K values are obtained according to Evanno et al. (2005). Results are based on the mean delta K of 10 replicates for each K. See Table 1 for information on sampling sites.


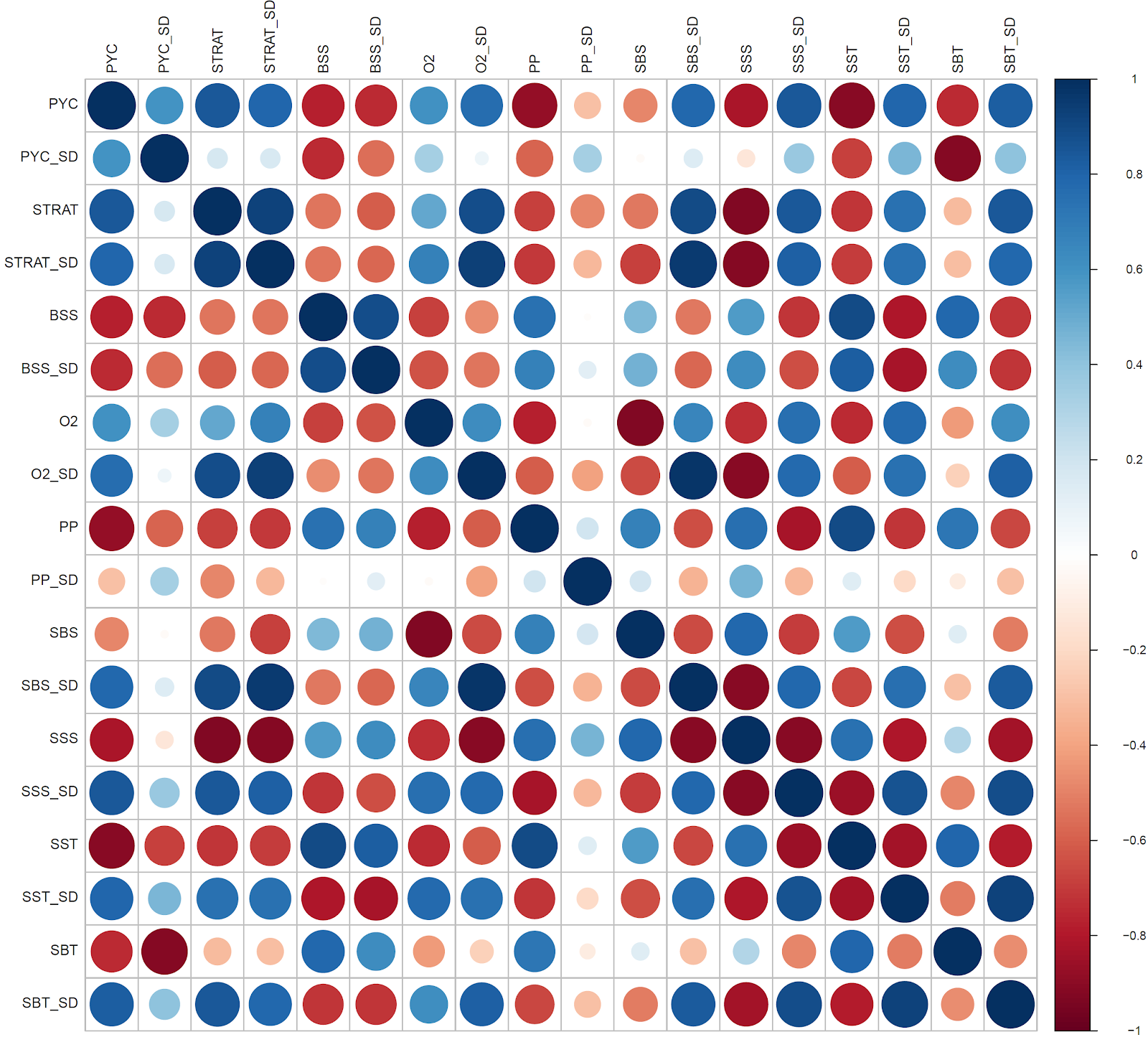


**Figure S2.** Correlation plot of the 18 environmental variables (ENV1) collected at the sampling sites of turbot. Forward selection following RDA identified an association between individual allele counts and variation in the depth of the pycnocline (PYC_SD), which correlates strongly with the sea bottom temperature (SBT; R = -0.92).


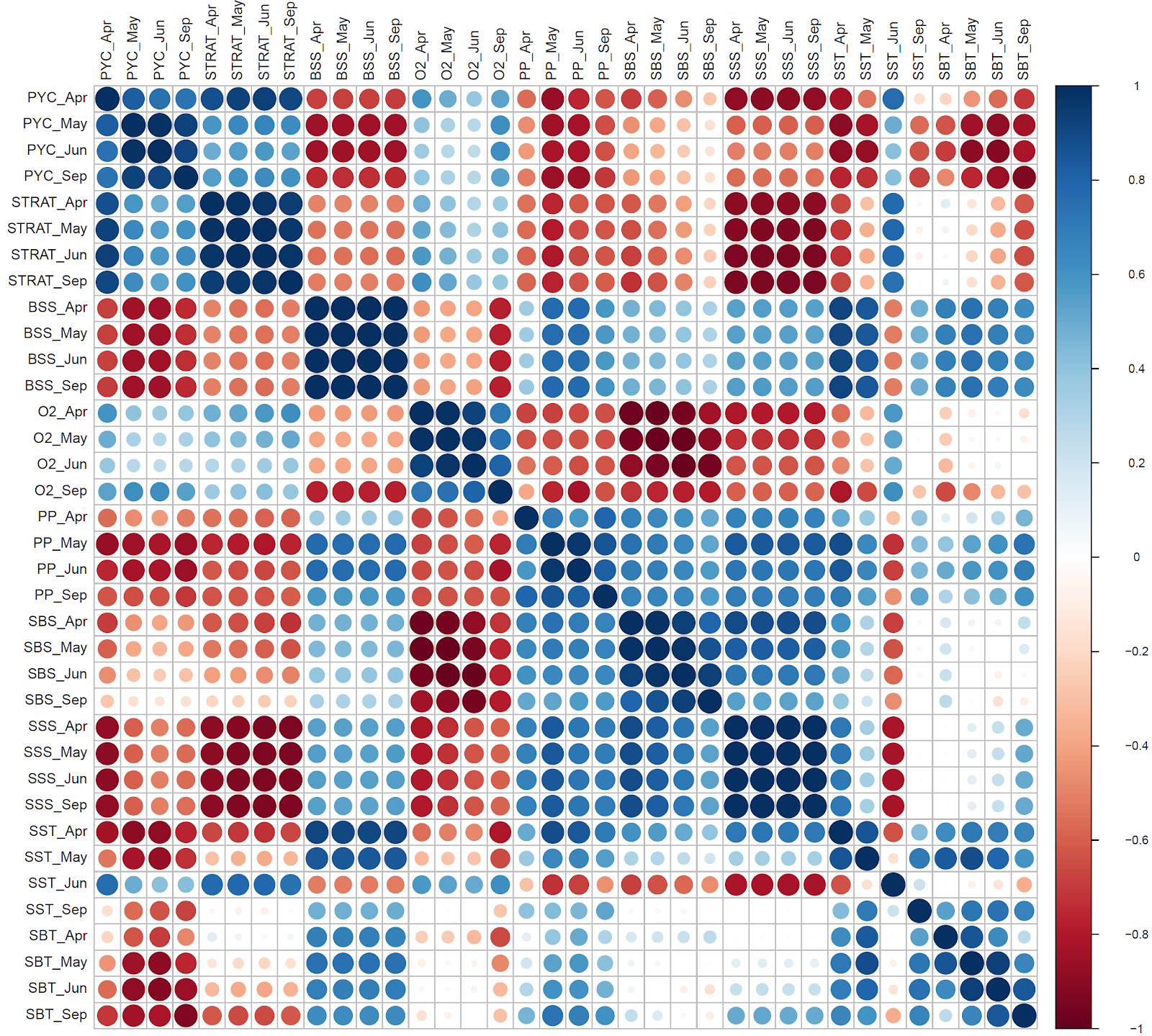


| **Figure S3.** Correlation plot of focal monthly environmental variables (ENV2) collected at the sampling sites of turbot. Forward selection following RDA identified an association between individual allele counts and sea surface temperature (SST_Sept), which did not strongly correlate with any other variable. Forward selection following RDA also identified an association between individual allele counts and sea bottom salinity in September (SBS_Sept), which correlates strongly with the sea bottom salinity in May and June (SBS_May; R = 0.86; SBS_Jun; R = 0.94), and with O2 in April, May and June (O2_Apr; R = -0.83; O2_May; R = -0.90; O2_Jun; R =  -0.96). |
| --- |


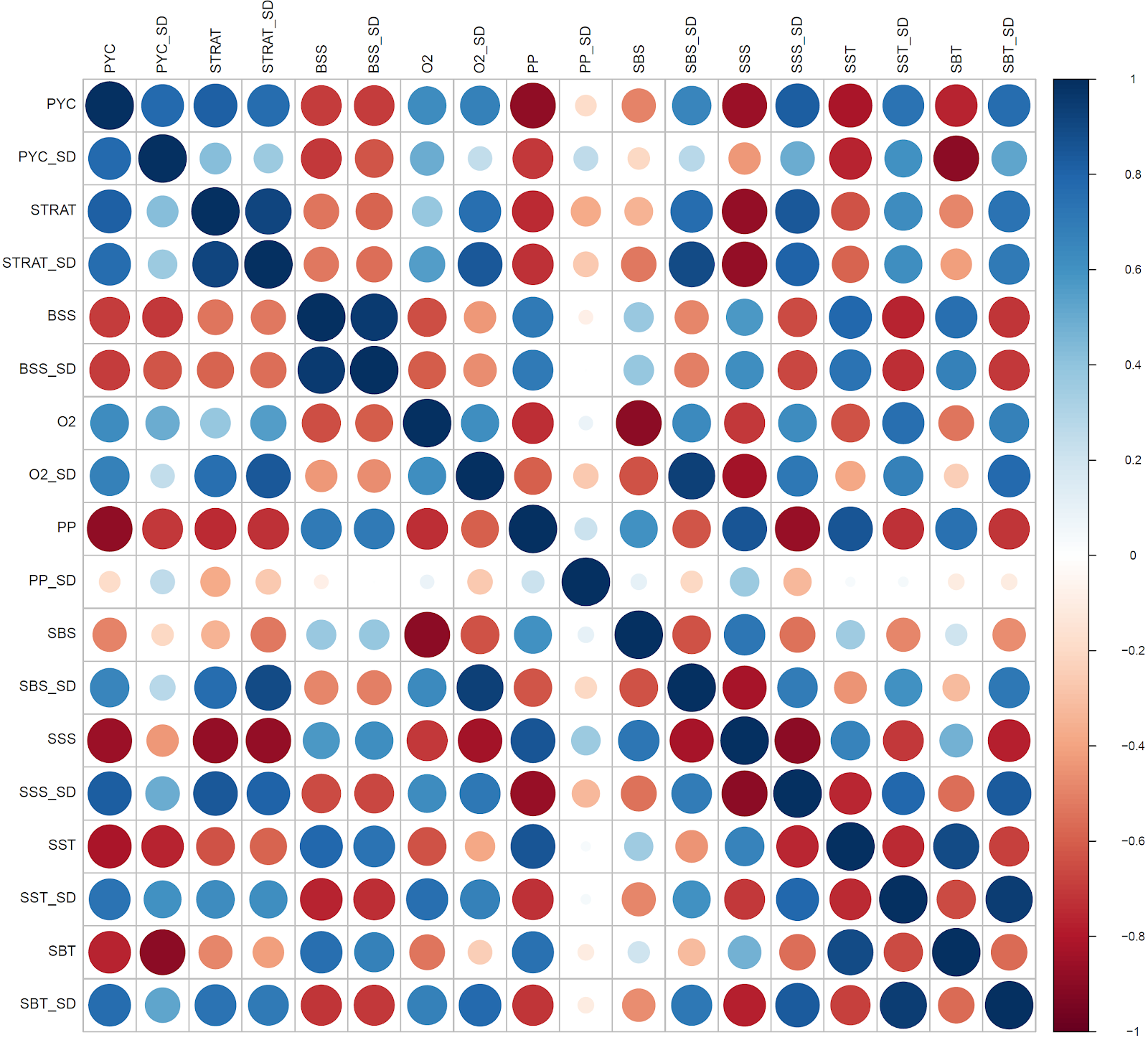


| **Figure S4.** Correlation plot of the 18 environmental variables (ENV1) collected at the sampling sites of brill. Forward selection following RDA identified an association between individual allele counts and stratification (STRAT), which correlates strongly with variation in stratification (STRAT_SD; R = 0.91), depth of pycnocline (PYC; R = 0.81), sea surface salinity (SSS; R = -0.88) and the variation in sea surface salinity (SSS_SD; R = 0.84). |
| --- |


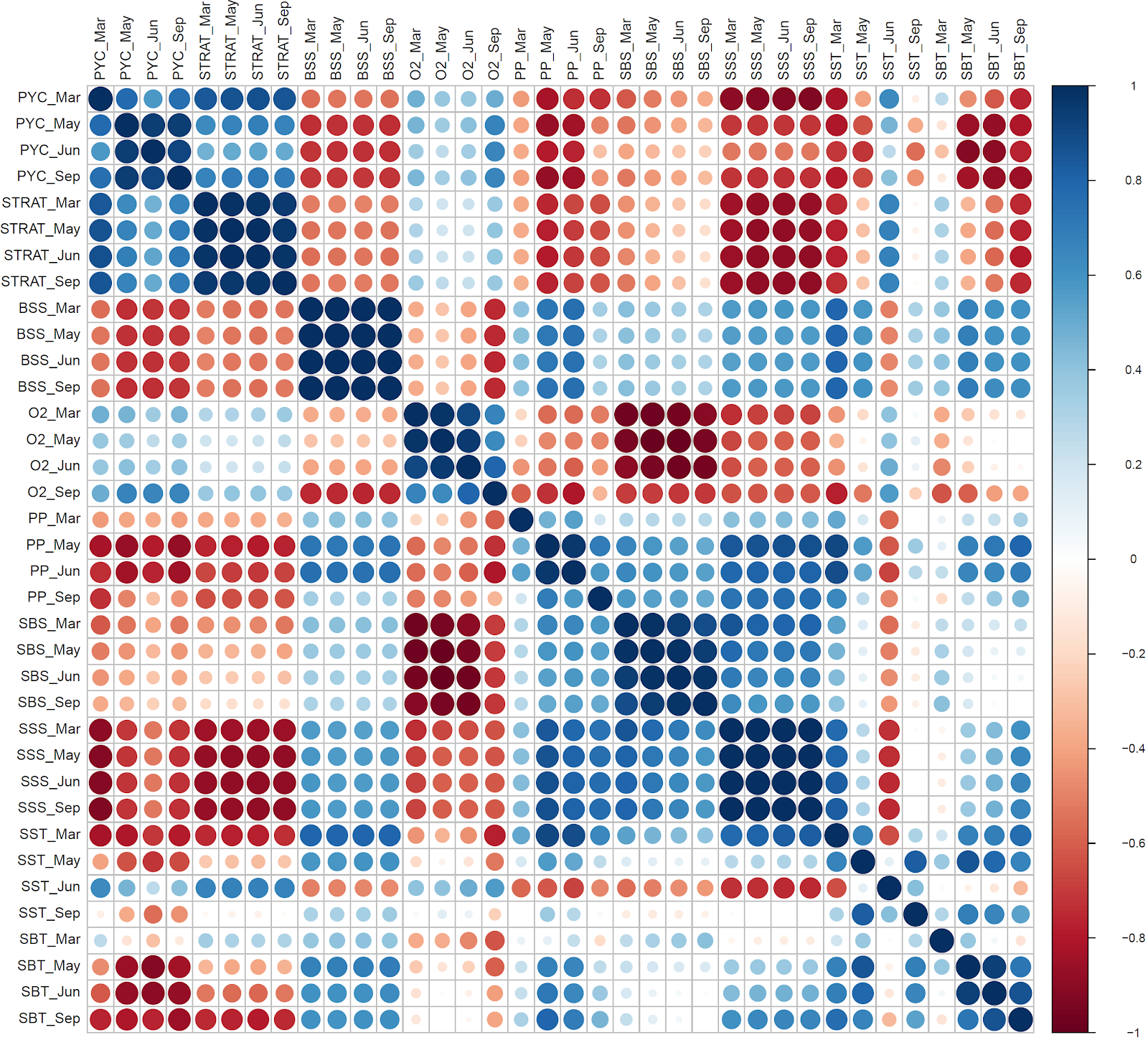


| **Figure S5.** Correlation plot of focal monthly environmental variables (ENV2) collected at the sampling sites of brill. Forward selection following RDA identified an association between individual allele counts and stratification in June (STRAT_Jun), which correlates strongly with the depth of pycnocline in March (PYC_Mar; R = 0.87), with stratification in March, May and September (STRAT_Mar; R = 0.97; STRAT_May; R = 0.99; STRAT_Sept = 0.98), and with sea surface salinity in March, May, June and September (SSS_Mar; R = -0.85; SSS_May; R = -0.89; SSS_Jun; R = -0.89; SSS_Sep; R = -0.88). |
| --- |


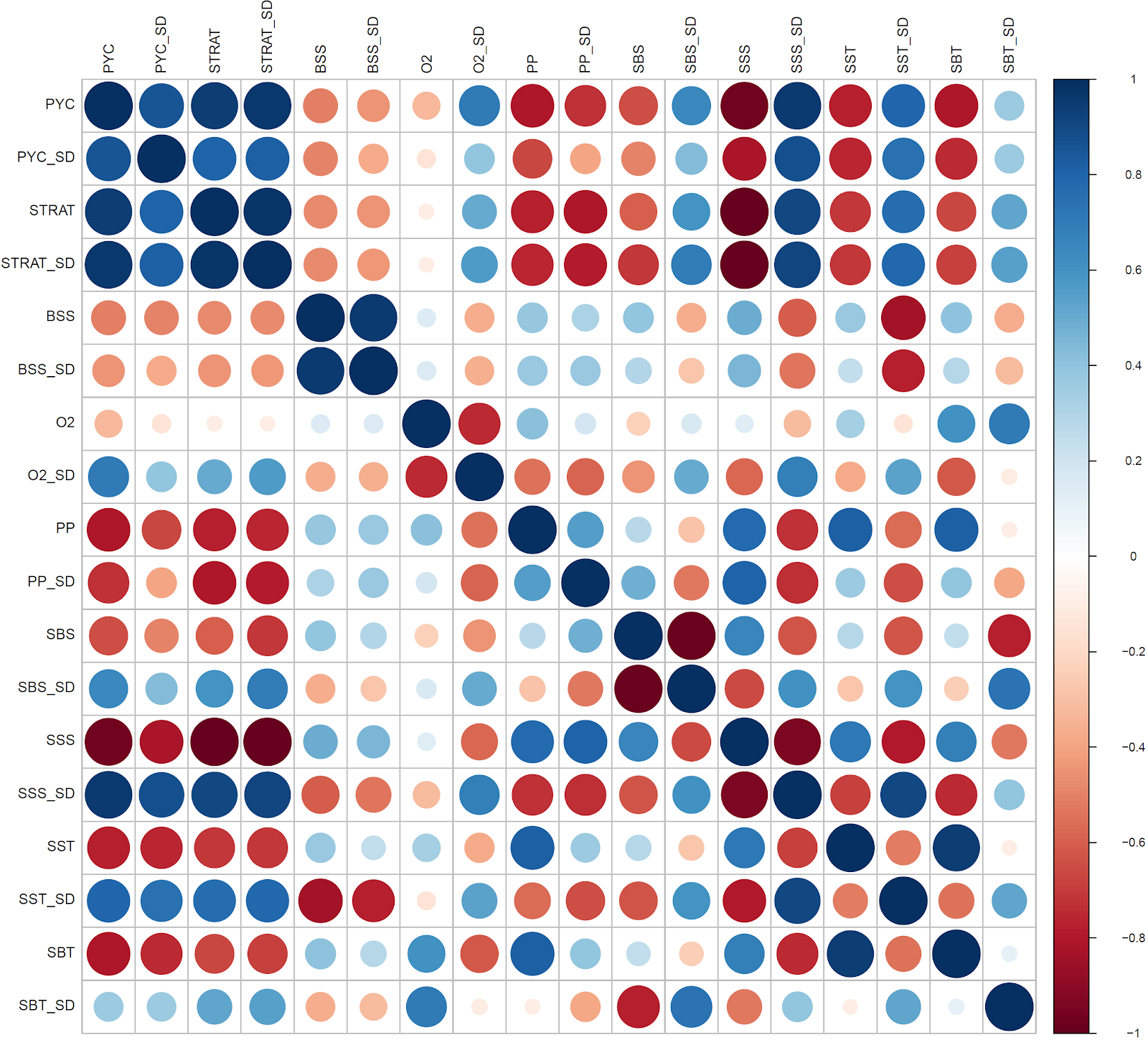


| **Figure S6.** Correlation plot of the 18 environmental variables (ENV1) collected at the sampling sites of sole. Forward selection following RDA did not identify an association between individual allele counts and any of the environmental variables. |
| --- |


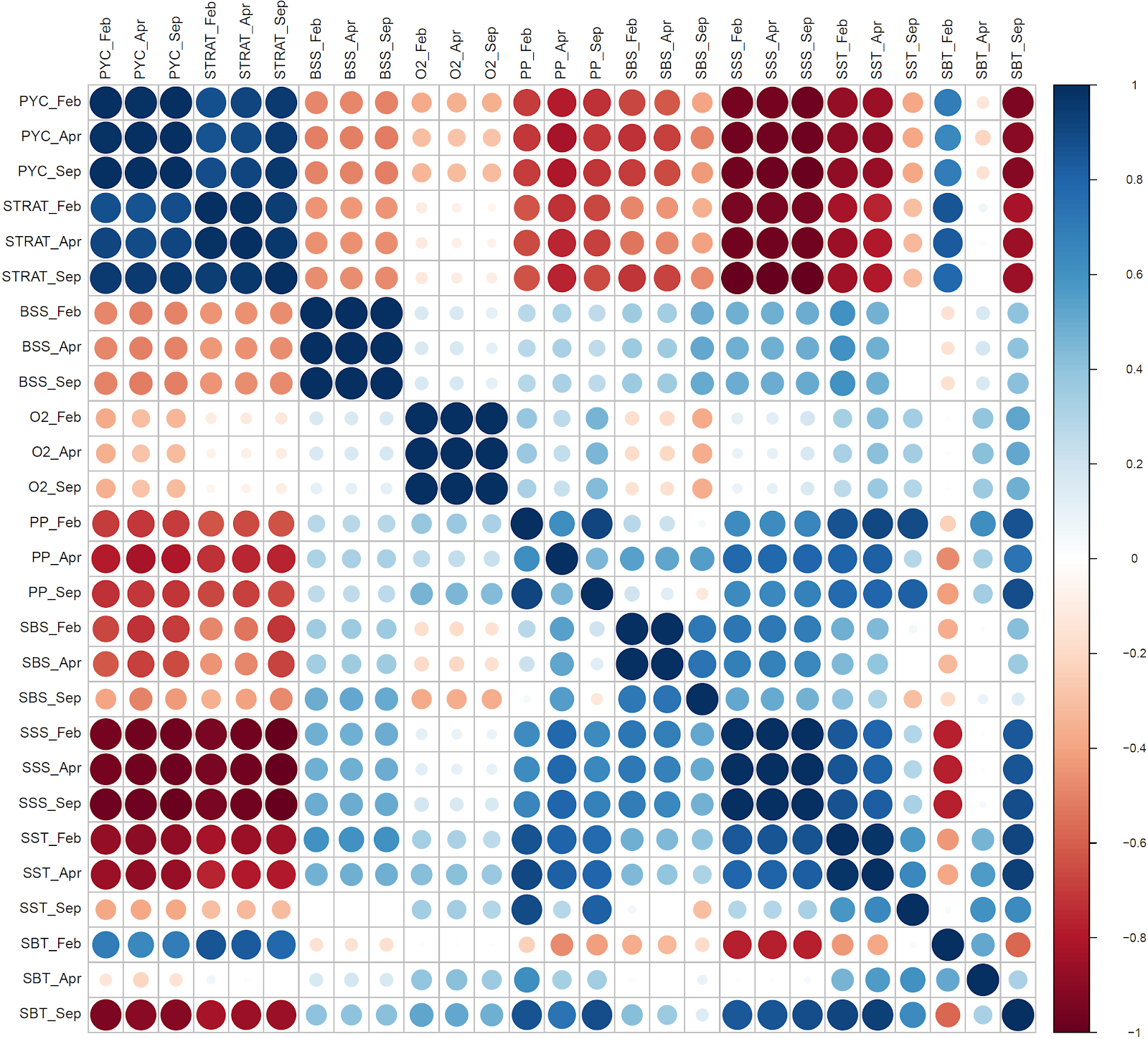


| **Figure S7.** Correlation plot of focal monthly environmental variables (ENV2) collected at the sampling sites of sole. Forward selection following RDA did not identify an association between individual allele counts and any of the environmental variables. |
| --- |


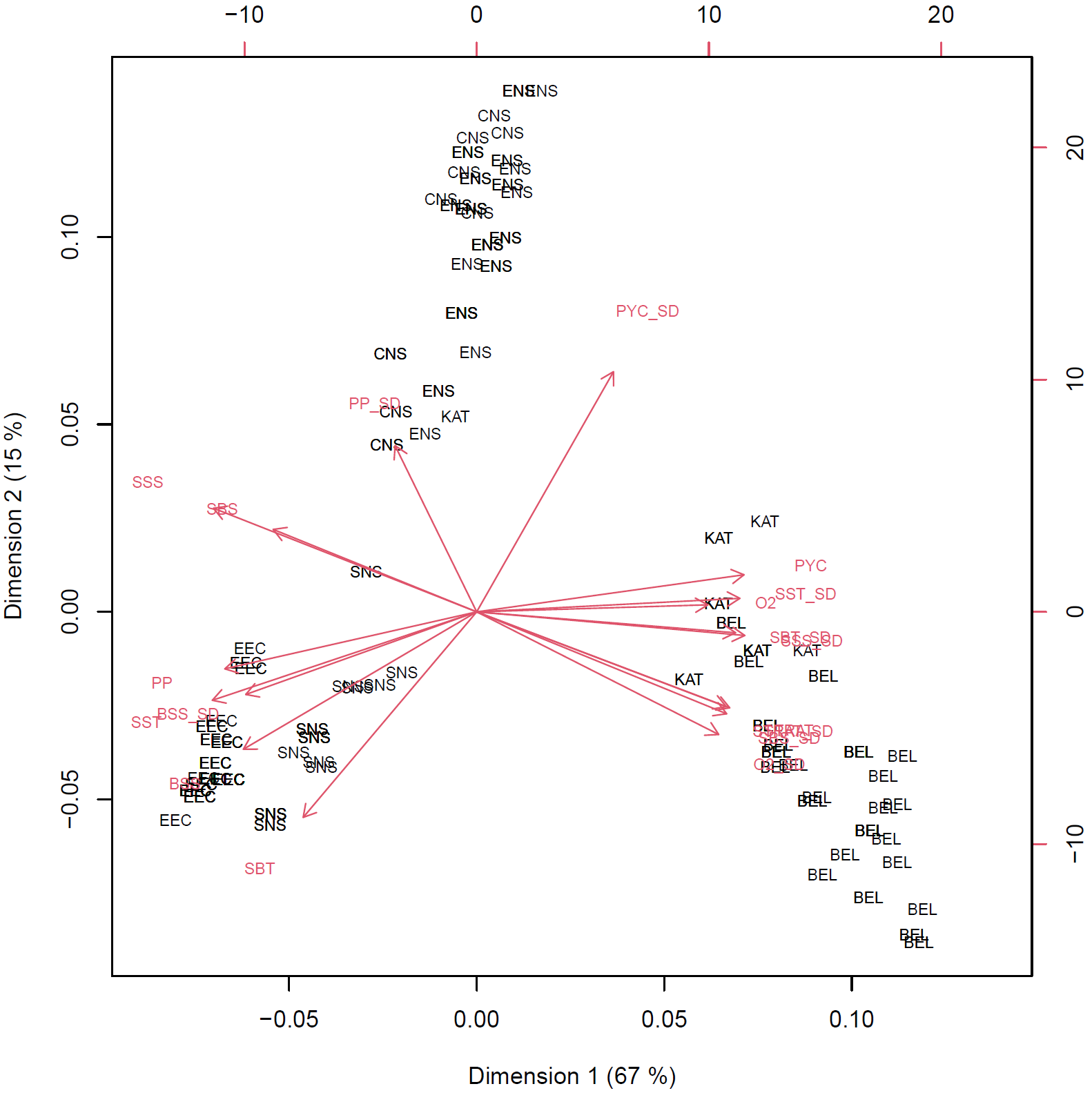


Figure S8. PCA analyses on 18 environmental variables collected at the sampling sites of turbot. Three clusters of sampling sites are identified: (1) sampling sites from the southern North Sea and the Channel (abbreviations: SNS and EEC), (2) sampling sites from the transition area (abbreviations: BEL and KAT), and (3) sampling sites from the German Bight and Central North Sea (abbreviations: ENS and CNS). Each arrow represents the yearly average or the standard deviation (SD) of an environmental variable. Abbreviations for the relevant variables are sea surface and sea bottom temperature (SST and SBT, respectively), salinity of the surface and bottom waters (SSS and SBS, respectively), bottom dissolved oxygen concentration (O_2_), net primary production (PP), bottom shear stress (BSS), depth of pycnocline (PYC) and stratification index (STRAT). For sampling site abbreviations see Table 2.


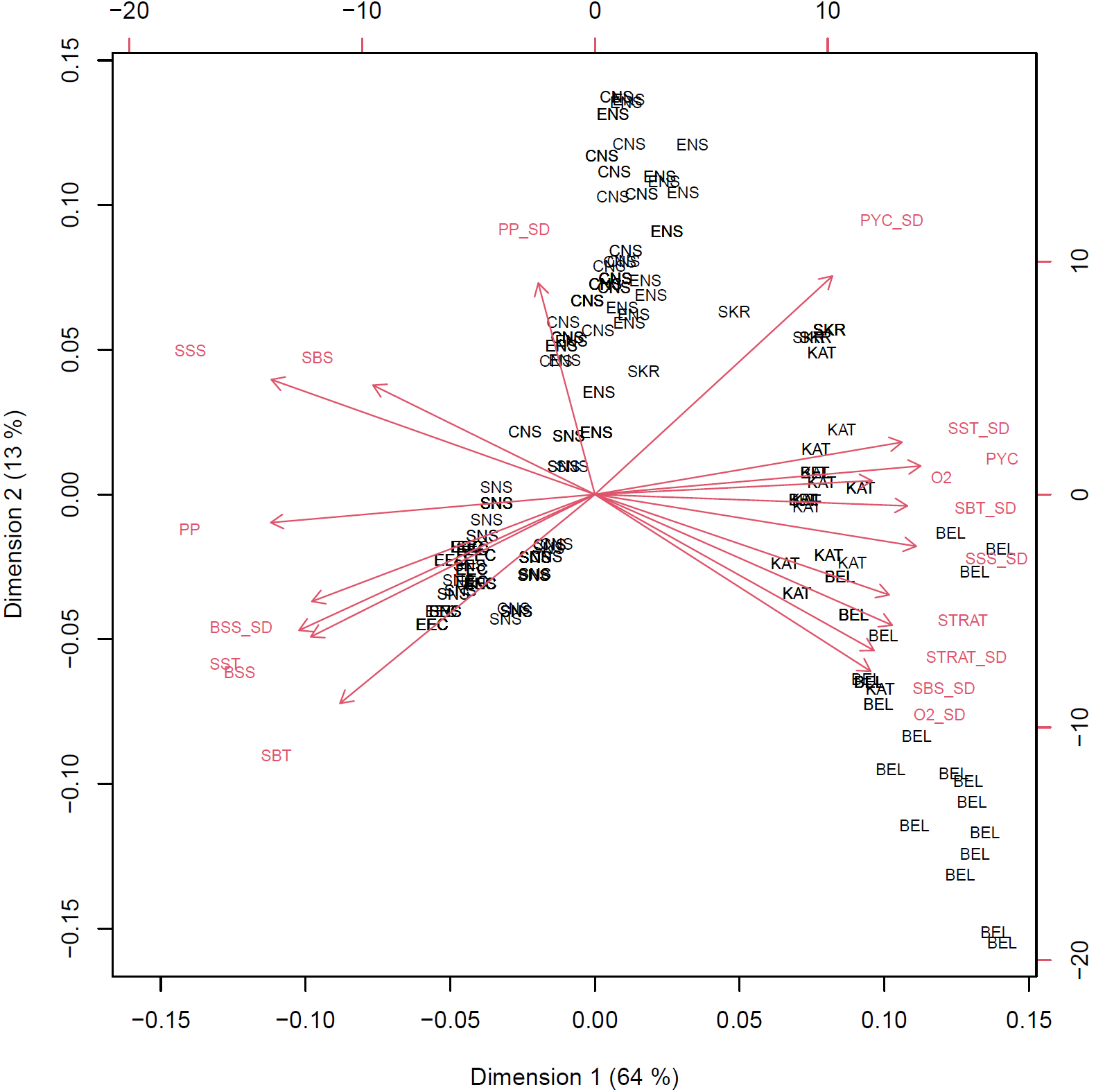


Figure S9. PCA analyses on 18 environmental variables collected at the sampling sites of brill. Three clusters of sampling sites are identified: (1) sampling sites from the southern North Sea and the Channel (abbreviations: SNS and EEC), (2) sampling sites from the transition area (abbreviations: BEL, KAT and SKR), and (3) sampling sites from the German Bight and Central North Sea (abbreviations: ENS and CNS). Each arrow represents the yearly average or the standard deviation (SD) of an environmental variable. Abbreviations for the relevant variables are sea surface and sea bottom temperature (SST and SBT, respectively), salinity of the surface and bottom waters (SSS and SBS, respectively), bottom dissolved oxygen concentration (O2), net primary production (PP), bottom shear stress (BSS), depth of pycnocline (PYC) and stratification index (STRAT). For sampling site abbreviations see Table 2.


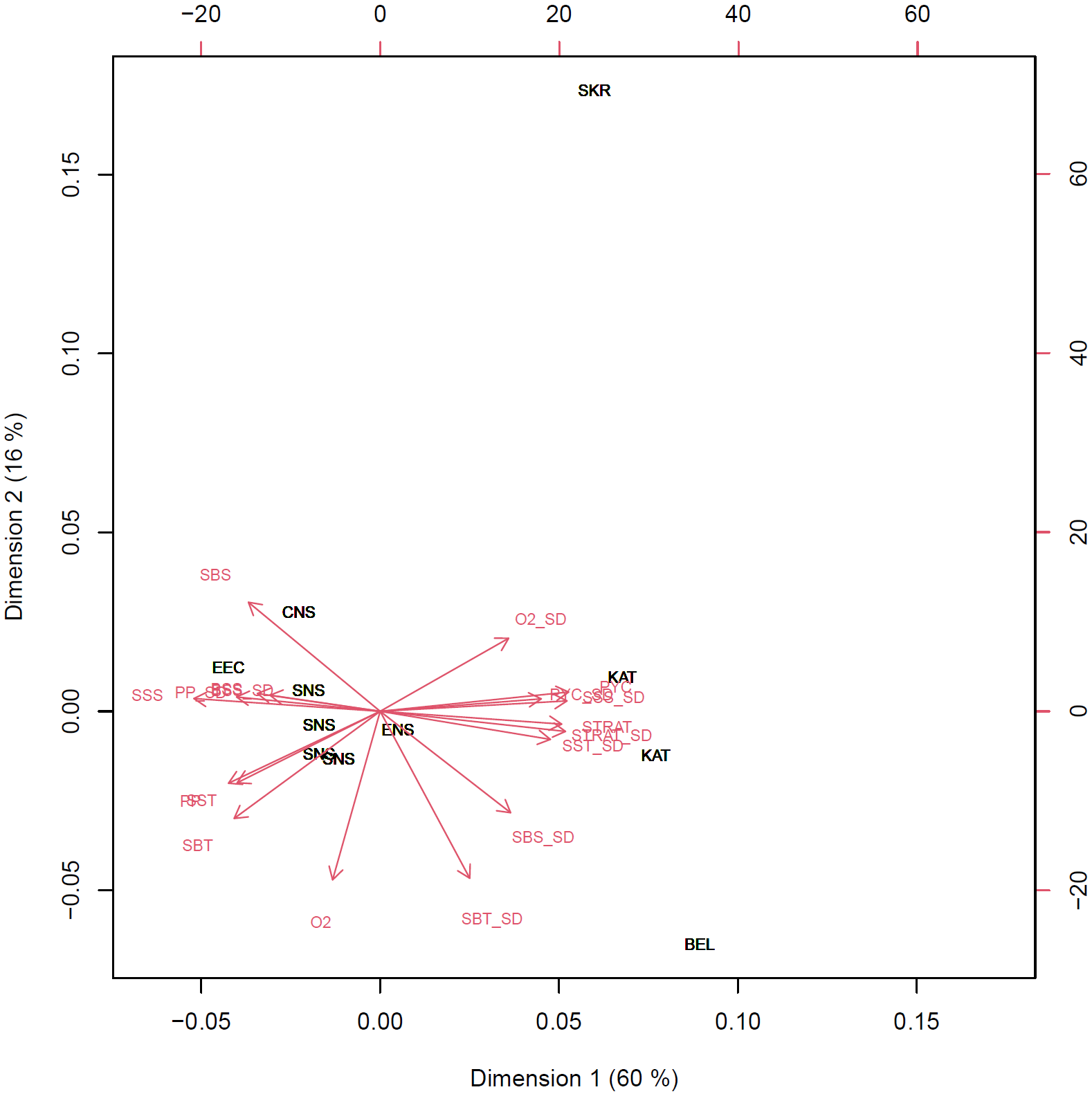


Figure S10. PCA analyses on 18 environmental variables collected at the sampling sites of sole. Two clusters of sampling sites are identified: (1) a cluster of sampling sites from the transition area (abbreviations: BEL and KAT), and (2) sampling sites from the Northeast Atlantic (abbreviations: ENS, CNS, SNS and EEC). Each arrow represents the yearly average or the standard deviation (SD) of an environmental variable. Abbreviations for the relevant variables are sea surface and sea bottom temperature (SST and SBT, respectively), salinity of the surface and bottom waters (SSS and SBS, respectively), bottom dissolved oxygen concentration (O_2_), net primary production (PP), bottom shear stress (BSS), depth of pycnocline (PYC) and stratification index (STRAT). For sampling site abbreviations see Table 2.


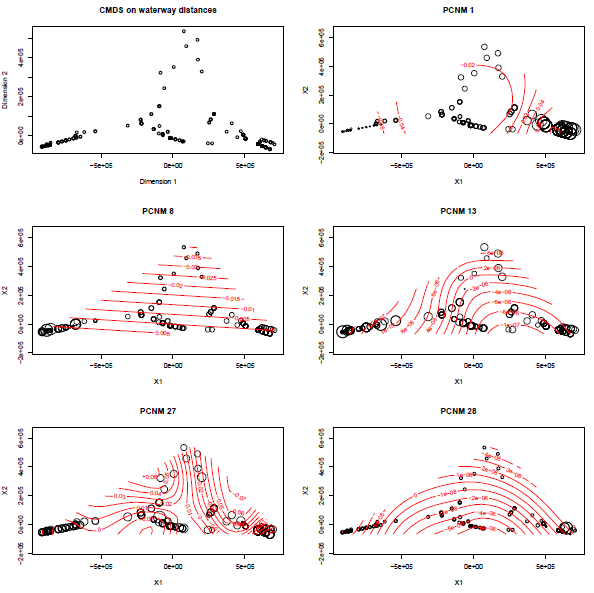


**Figure S11.** Graphic representation of the selected MEMs for turbot based on a distance matrix calculated on waterway distance between species-specific sampling locations.


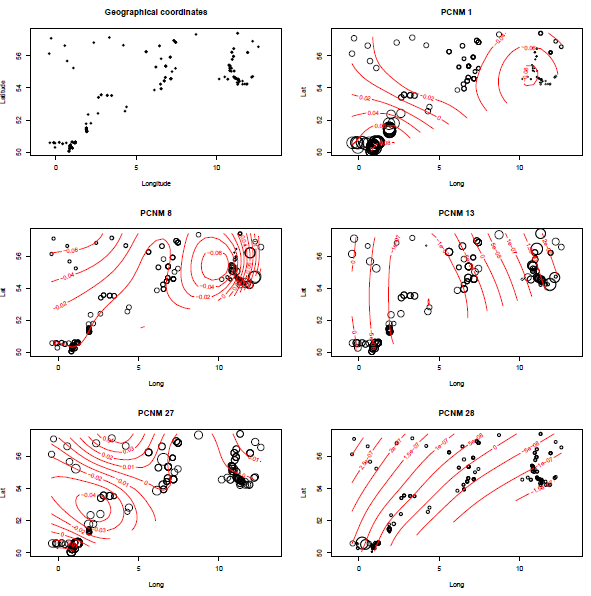


**Figure S12.** Graphic representation of the selected MEMs for turbot based on a distance matrix calculated on geographic distance between species-specific sampling locations.


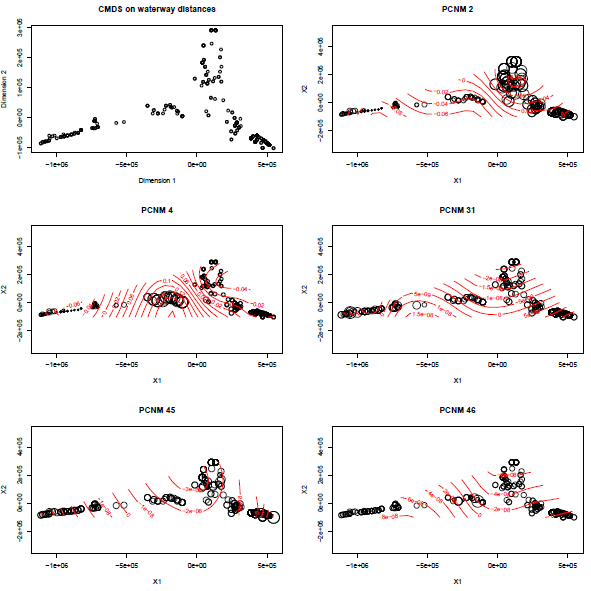


Figure S13. Graphic representation of the selected MEMs for brill based on a distance matrix calculated on waterway distance between species-specific sampling locations.


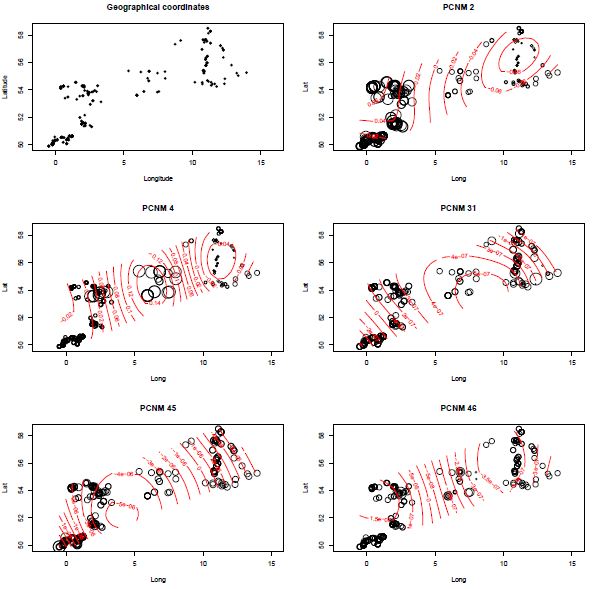


**Figure S14**. Graphic representation of the selected MEMs for brill based on a distance matrix calculated on geographic distance between species-specific sampling locations.
